# Supplementary figures and images for: Hierarchical recurrent temporal prediction as a model of the mammalian dorsal visual pathway
Source: PLoS Comput Biol. 2026 May 28;22(5):e1013138. doi: 10.1371/journal.pcbi.1013138 (PMC13252843; doi:10.1371/journal.pcbi.1013138)

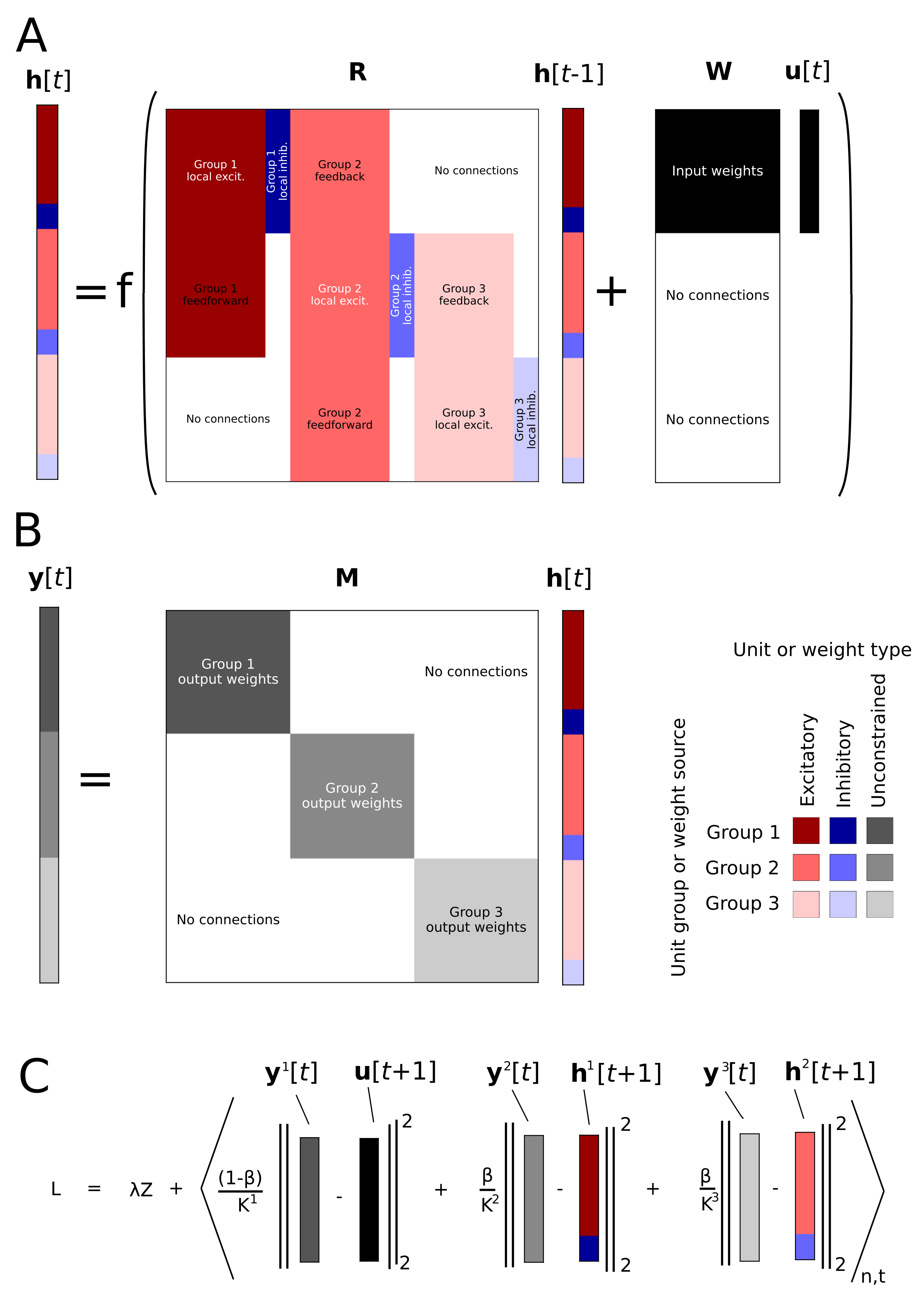

Supplement: S1 Fig — The squares and broad vertical rectangles illustrate the weight matrices. The shade (dark/mid/light) indicates what group (G1/G2/G3) the weights in that region of the matrix project from. The color (white/red/blue/gray) indicates whether those weights are constrained to be zero (white), positive (red), negative (blue) or unconstrained (gray). The narrow vertical rectangles illustrate the vectors, with shade indicating group and color indicating whether they are excitatory units (red), inhibitory units (blue) or unconstrained output units (gray). Vectors have been widened slightly for visualization purposes only. Bias vectors have been left off for simplicity. (A) Illustration of the equation for the hidden unit activity (Equation 1). This shows the input vector, u[t], the input weight matrix, W, the hidden unit vector, h[t], and the recurrent weight matrix, R. Note the inhibitory (blue) and excitatory (red) units of the hidden unit vector and their corresponding constrained weights in the recurrent matrix. (B) Illustration of the equation for the network output (Equation 2). This shows the output vector, y[t], and output weight matrix, M, along with the hidden unit vector to which the output weights are applied. (C) Illustration of the loss function (Equation 3), using the input vector and groups (h1[t], h2[t], y1[t], y2[t], y3[t]) from the hidden and output vectors. (TIFF) [file pcbi.1013138.s001.tiff]

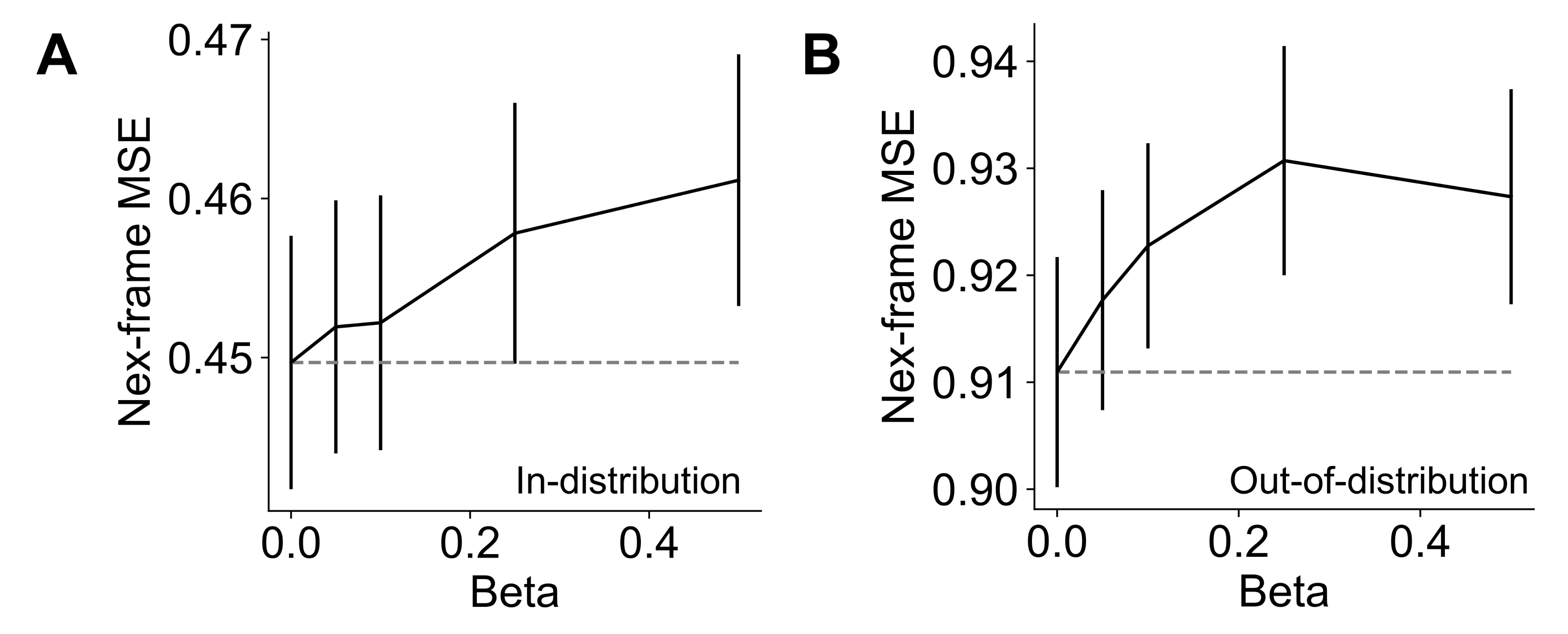

Supplement: S2 Fig — Next-frame prediction error increased as a function of beta for both the in-distribution (A) and out-of-distribution datasets (B). (TIFF) [file pcbi.1013138.s002.tiff]

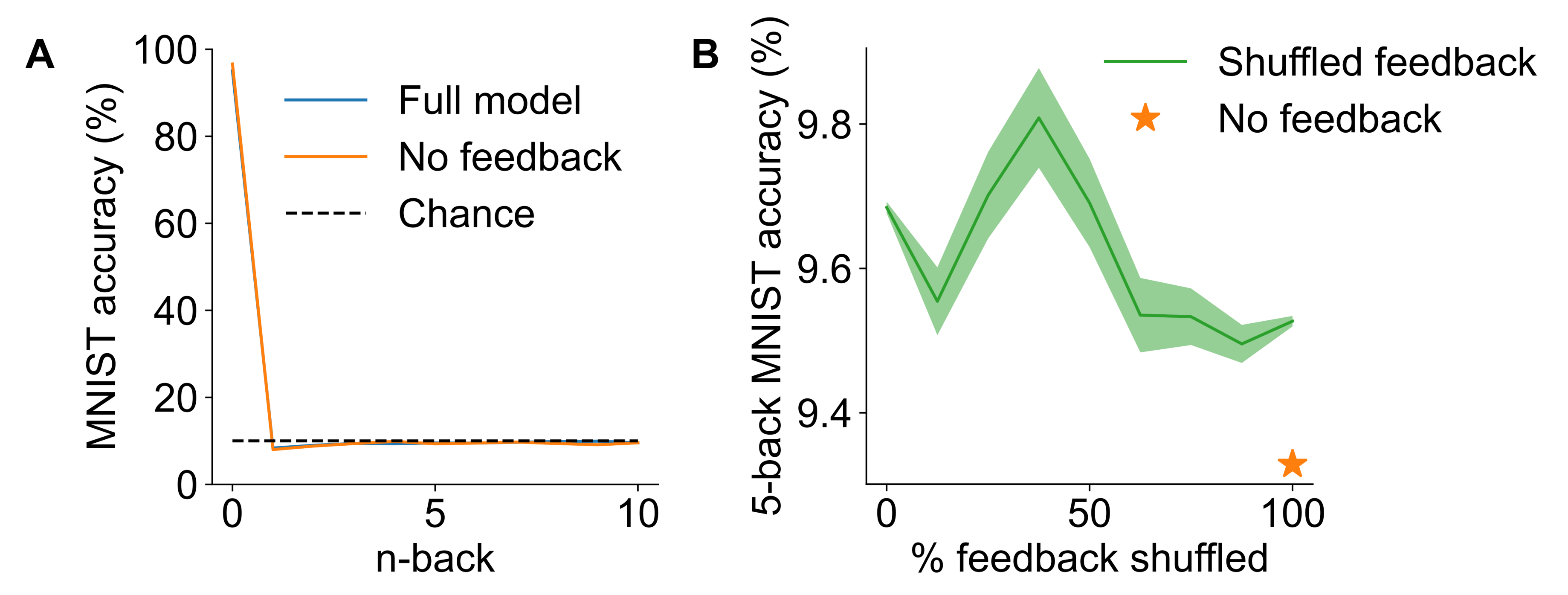

Supplement: S3 Fig — Here we trained a 0-back decoder and tested this fixed decoder for each n-back condition. We found that the decoder – in both the full and no feedback conditions, as well the shuffled feedback condition – was unable to generalize from the 0-back condition and performed at chance. Given the performance of the model in Fig 1J-1K when trained and tested on the same n-back condition (with separate train and test splits), it is unlikely that these fixed 0-back decoder results reflect an absence of information in the network over time. Rather, this suggests that using a fixed decoder is not sufficient and that it needs to be re-trained for each n-back condition to extract the information, as we do in Fig 1J-1K. (A) MNIST accuracy declines to chance at decoding from the n-back condition if a fixed 0-back decoder is used, for both the full and no feedback models. (B) MNIST accuracy at 5-back performs at chance as the percentage of feedback is shuffled for a fixed 0-back decoder. In both cases, chance performance indicates that the decoder is unable to generalize from the 0-back condition. (TIFF) [file pcbi.1013138.s003.tiff]

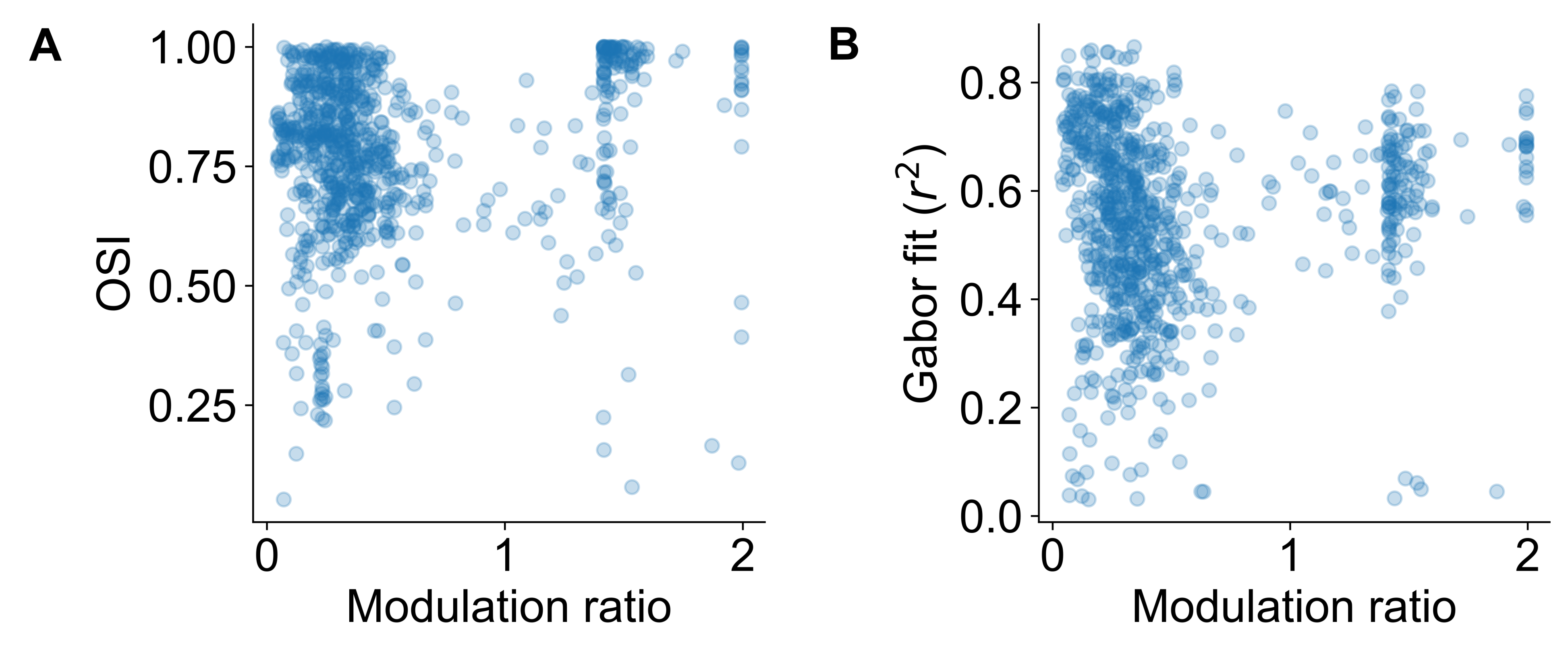

Supplement: S4 Fig — The simple-cell-like units (modulation ratio > 1) had higher mean orientation selectivity (A) and were better fit by the Gabor function (B) than the complex-cell-like units. (TIFF) [file pcbi.1013138.s004.tiff]

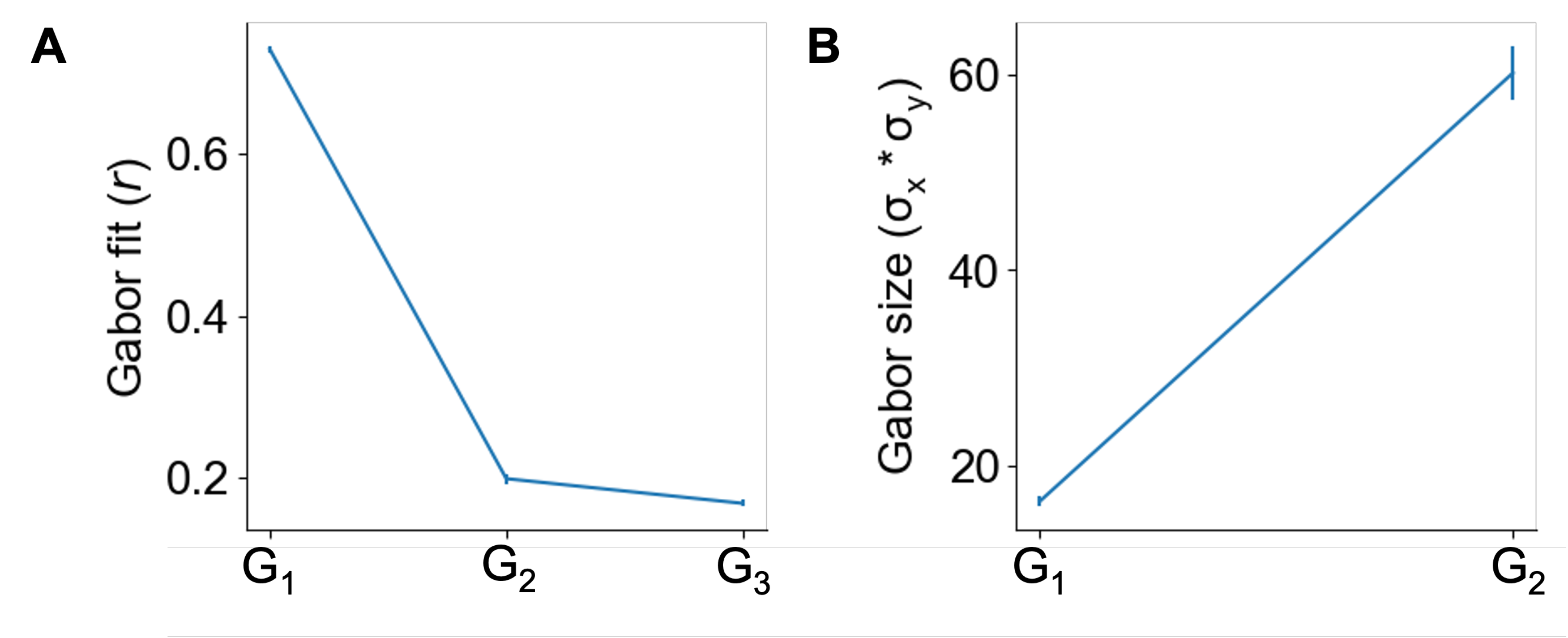

Supplement: S5 Fig — (A) Only units in the model’s first group could be modeled as Gabors, with higher groups showing a poorer fit (Gabor r for G1 vs G2: t(16.6)=7.43, p < 0.0001). (B) For the subset of units that could be modeled as a Gabor (r > 0.3), the receptive field size – indexed by the product of the x and y Gabor standard deviation parameters – increased from group 1 to group 2 (Gabor size for G1 vs G2: t(7.04)=-4.22, p = 0.004). (TIFF) [file pcbi.1013138.s005.tiff]

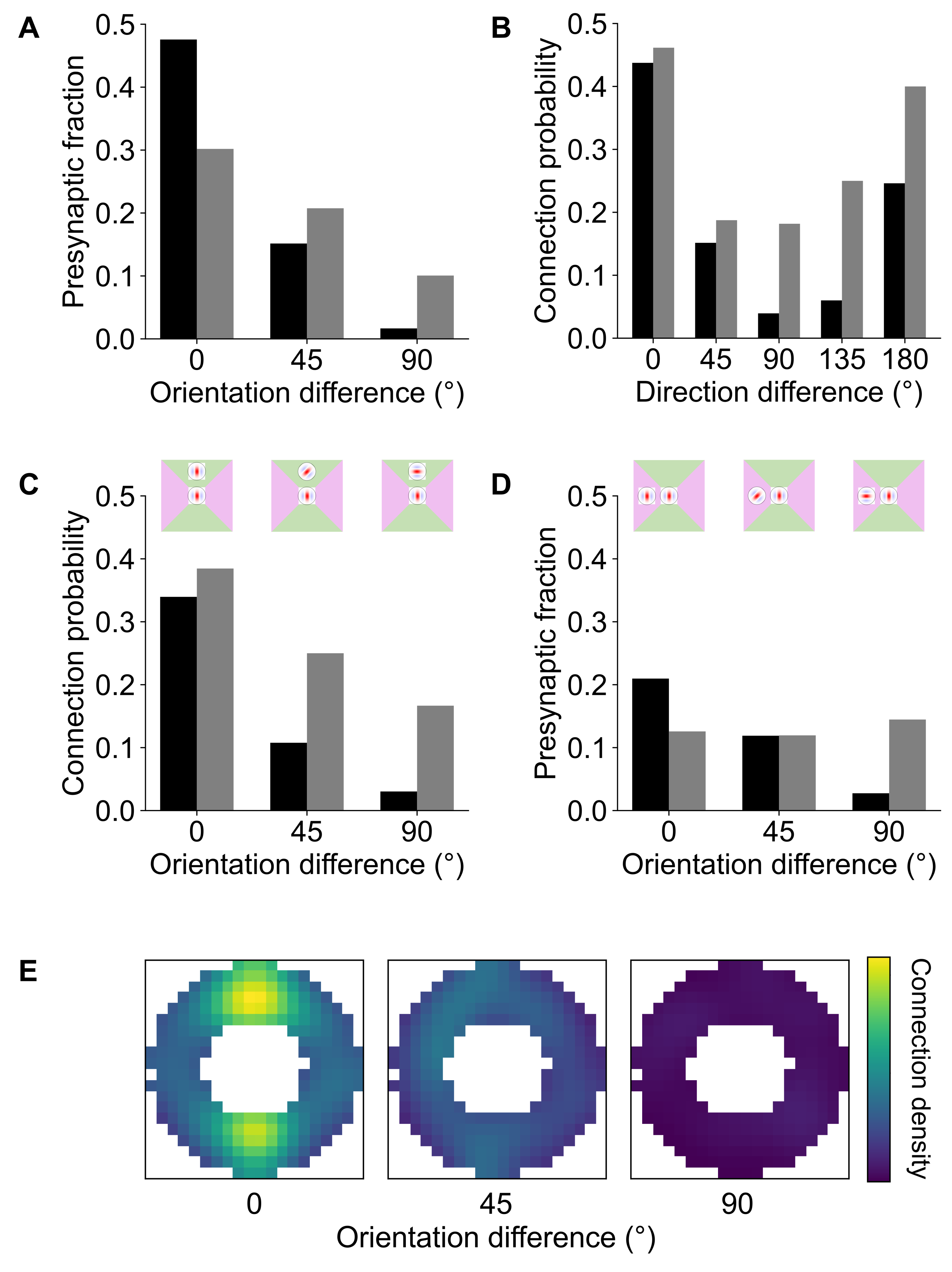

Supplement: S6 Fig — (A-B) Short-range connections between model units (black bars) are more prevalent for excitatory units that have similar orientation tuning (A) and direction-tuned units that have similar or opposite preferred directions of motion (B), as is also the case in V1 neurons (gray bars) [31]. (C-E) In both the model and V1 [32], long-range connection probability is higher for units with similar orientation preferences when their receptive fields are located in co-axial (C) than in co-orthogonal (D) locations. Heatmap (E) shows the normalized connection probability over visual space (relative to postsynaptic neuron position) across differences in orientation tuning for model units. For detailed methods, see Klavinskis-Whiting et al. [6]. (TIFF) [file pcbi.1013138.s006.tiff]

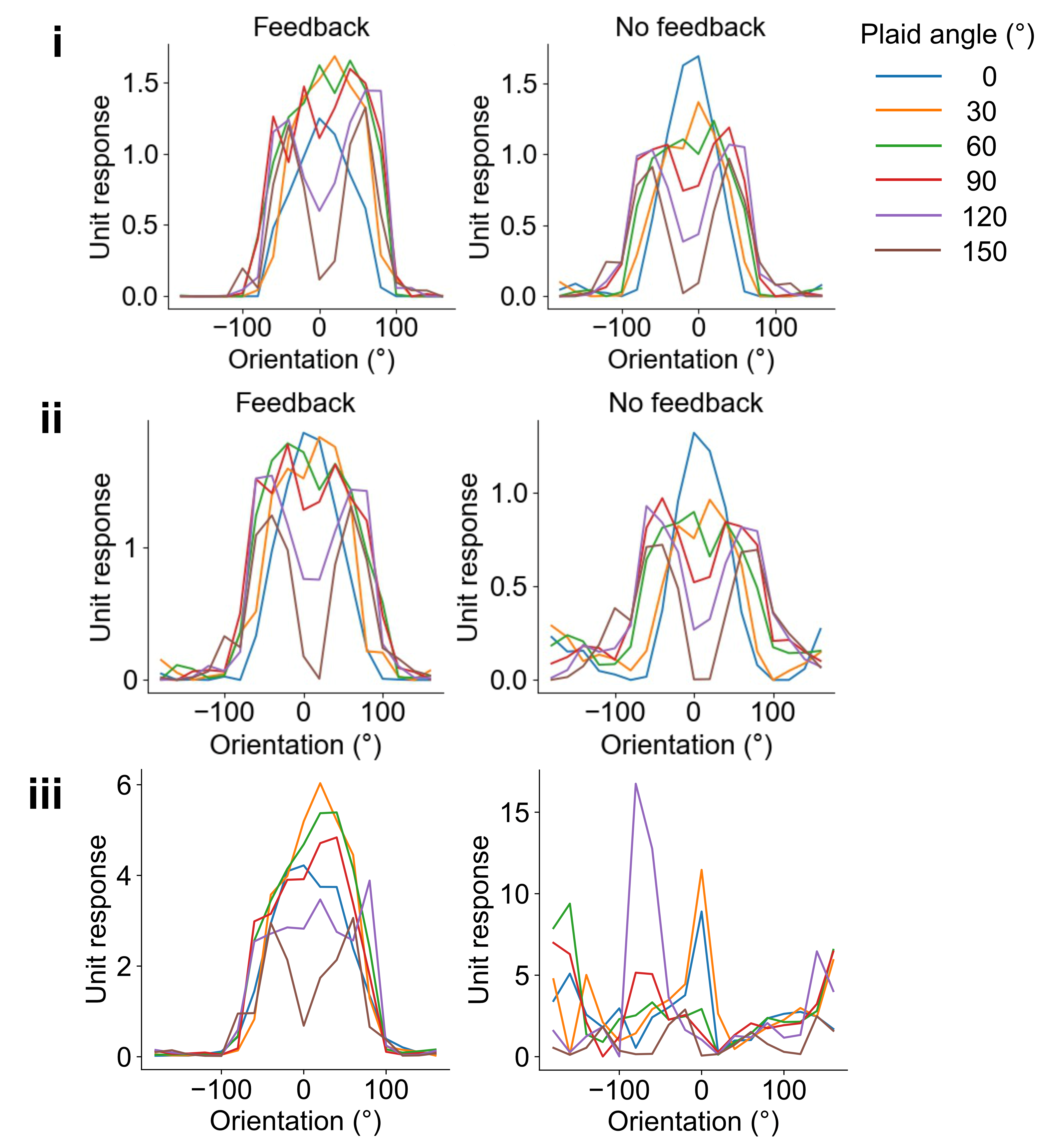

Supplement: S7 Fig — Direction tuning curves for plaid stimuli across different plaid angles are shown for three example model units with (left) and without (right) feedback. (TIF) [file pcbi.1013138.s007.tif]

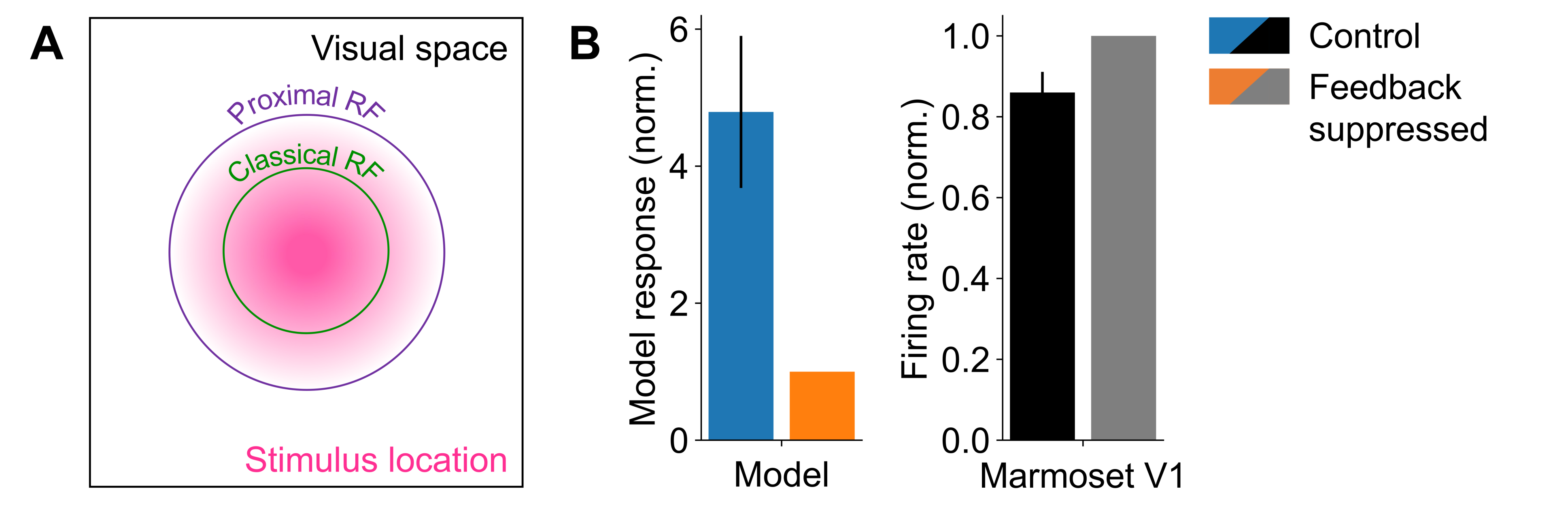

Supplement: S8 Fig — (A) Diagram of the classical and proximal receptive field for an exemplar neuron. (B) The full model’s average response rate is higher for stimuli spanning the classical and proximal receptive field than when feedback is suppressed. This contrasts with the data from marmoset V1, where the opposite trend is observed, such that the average neural response for stimuli spanning the classical and proximal receptive field is greater when feedback is suppressed [13]. (TIFF) [file pcbi.1013138.s008.tiff]

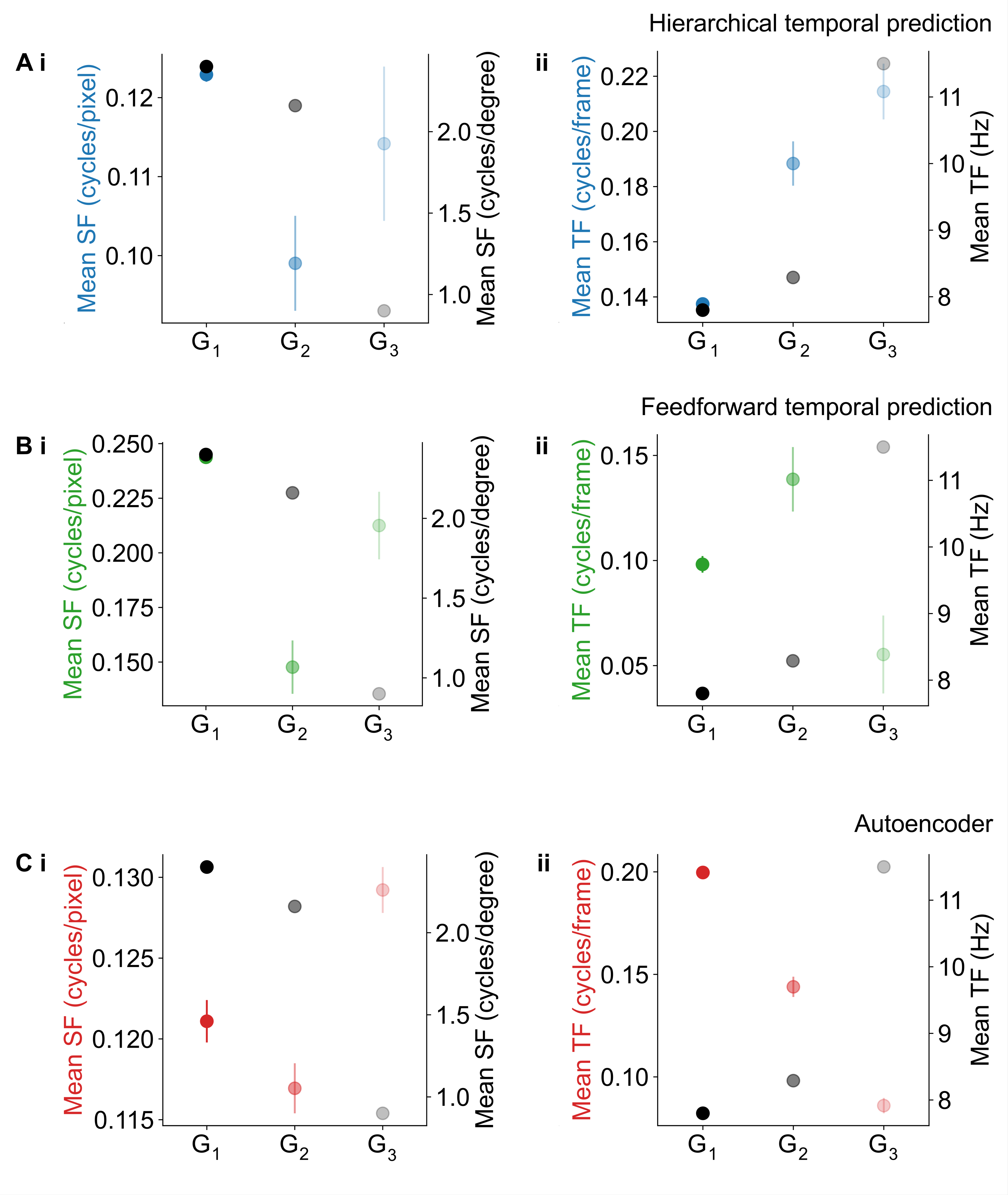

Supplement: S9 Fig — Spatial frequency tuning (i) and temporal frequency tuning (ii) across the (A) hierarchical recurrent temporal prediction model (blue), (B) feedforward temporal prediction model (green) and (C) autoencoder (red) models compared with data from the macaque visual cortex (gray). Experimental data as in Fig 2F,H. (TIFF) [file pcbi.1013138.s009.tiff]

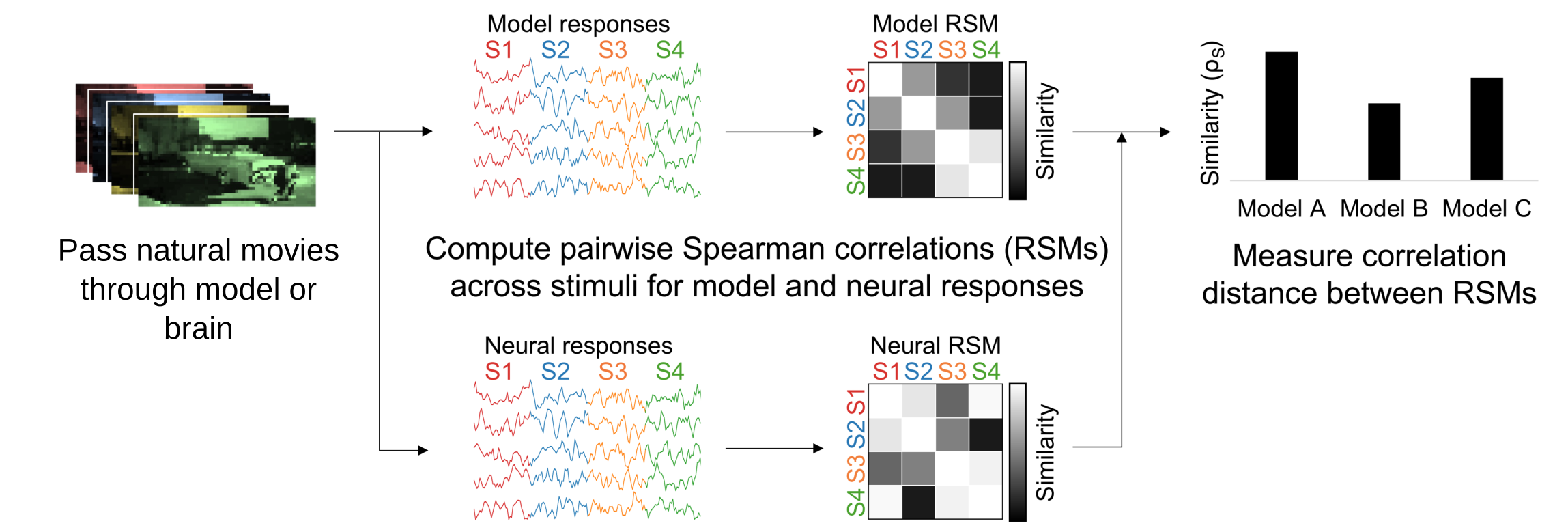

Supplement: S10 Fig — (TIFF) [file pcbi.1013138.s010.tiff]

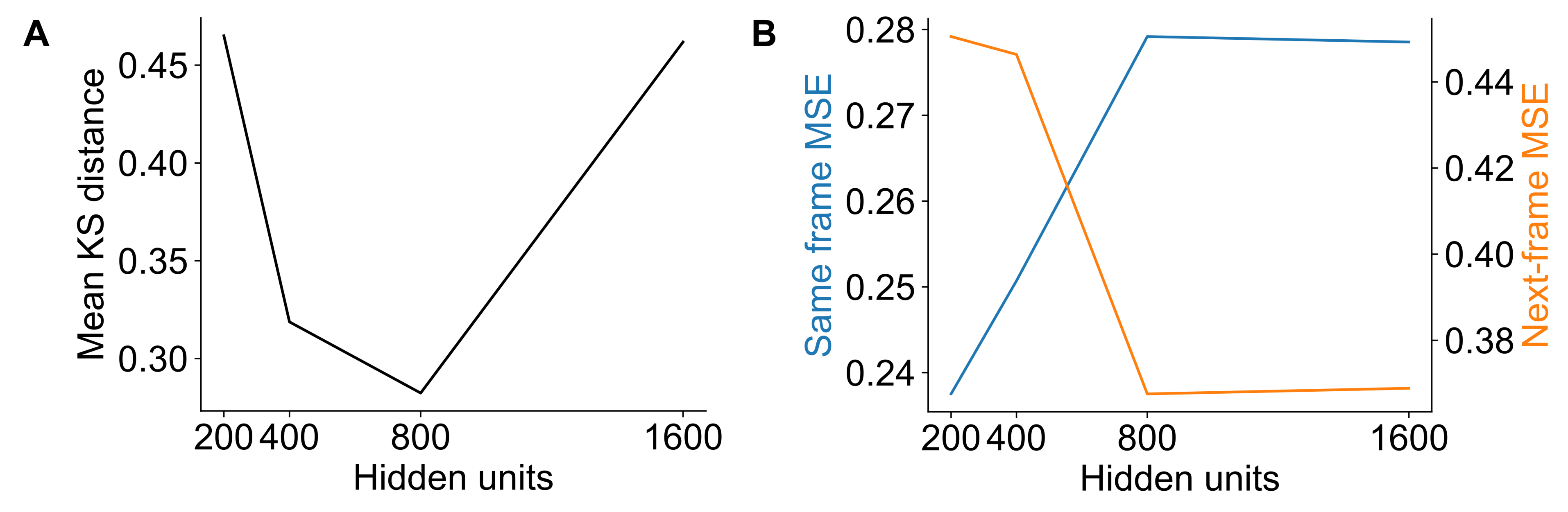

Supplement: S11 Fig — (A) Mean KS distance between model and neural distributions was lowest at 800 units per group. (B) As the number of hidden units increased, the model shifted from representing the current frame (same frame MSE, which increased as the number of hidden units increased) to predicting the upcoming frame (next-frame MSE, which decreased as the number of hidden units increased). Thus, a larger number of hidden units improved the network’s capacity for temporal prediction. (TIFF) [file pcbi.1013138.s011.tiff]

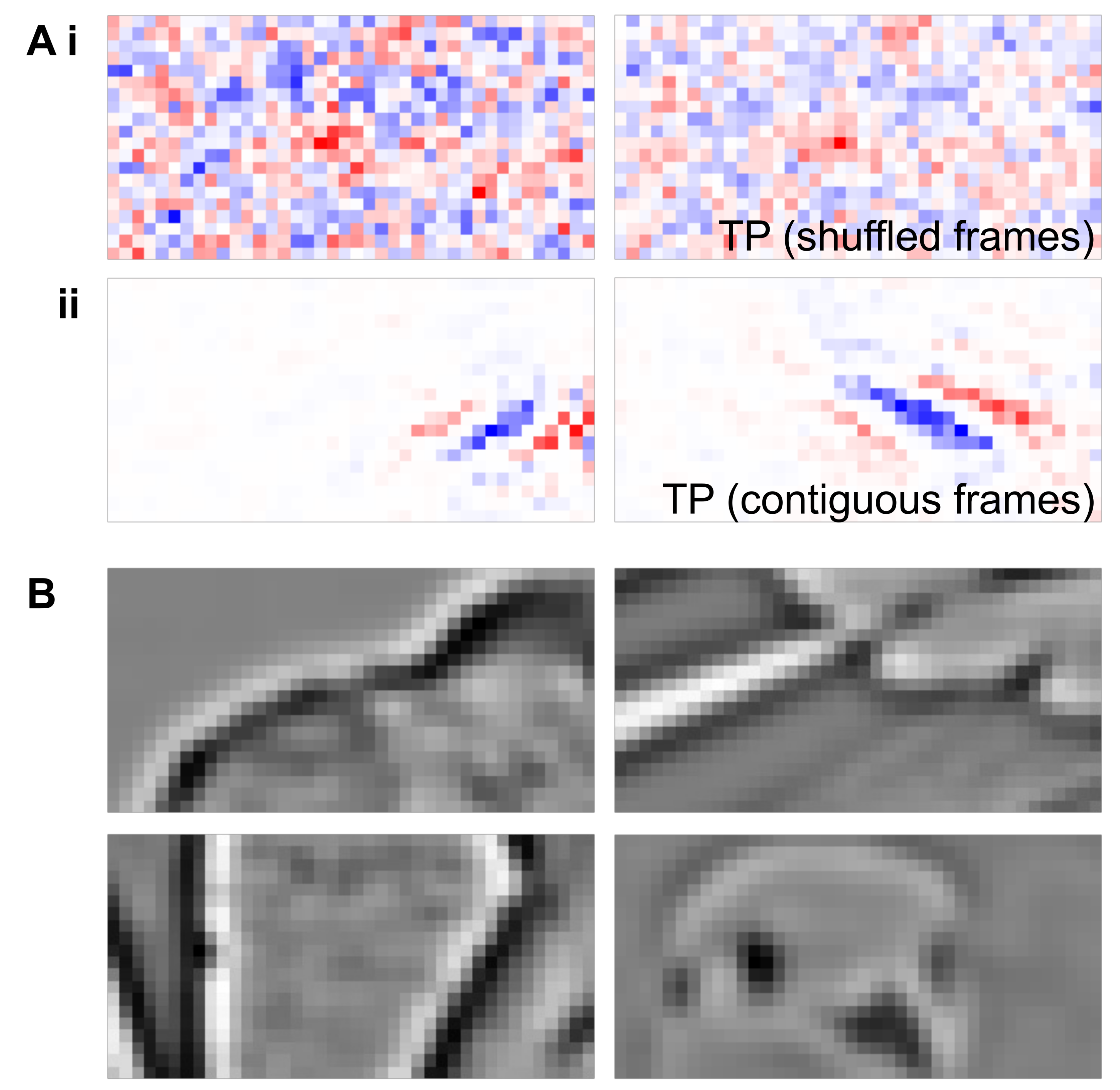

Supplement: S12 Fig — (A) A network trained on the same dataset but with temporal contiguities removed by shuffling the frame order fails to produce coherent receptive fields (i). Conversely, the standard temporally-contiguous dataset produces receptive fields with a clear Gabor-like structure (ii). (B) Example frames from the dataset used for model training. (TIFF) [file pcbi.1013138.s012.tiff]

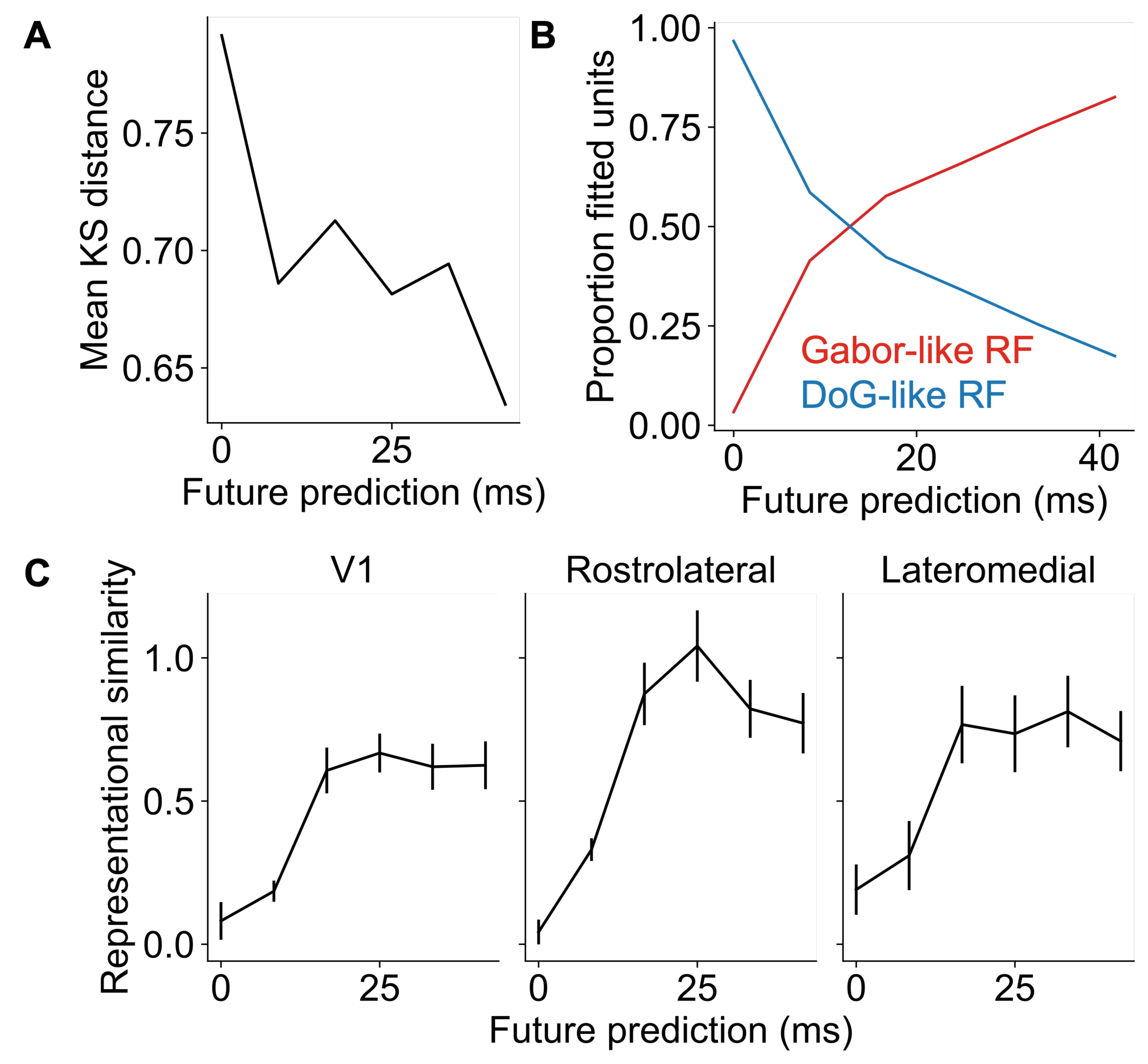

Supplement: S13 Fig — (A) The mean KS distance between the model and brain distributions of modulation ratios, plaid pattern indices and surround suppression indices. A lower score indicates a more brain-like distribution in the model, with the autoencoder (0 ms temporal offset/future prediction) having the least brain-like distribution. (B) The distribution of Gabor-like versus difference-of-Gaussian-like units in model group 1 (G1) across temporal offsets, with the highest proportion of Gabor-like units found when predicting the next frame 42 ms into the future. (C) Representational similarity between the model and different regions of mouse visual cortex across temporal offsets. (TIFF) [file pcbi.1013138.s013.tiff]

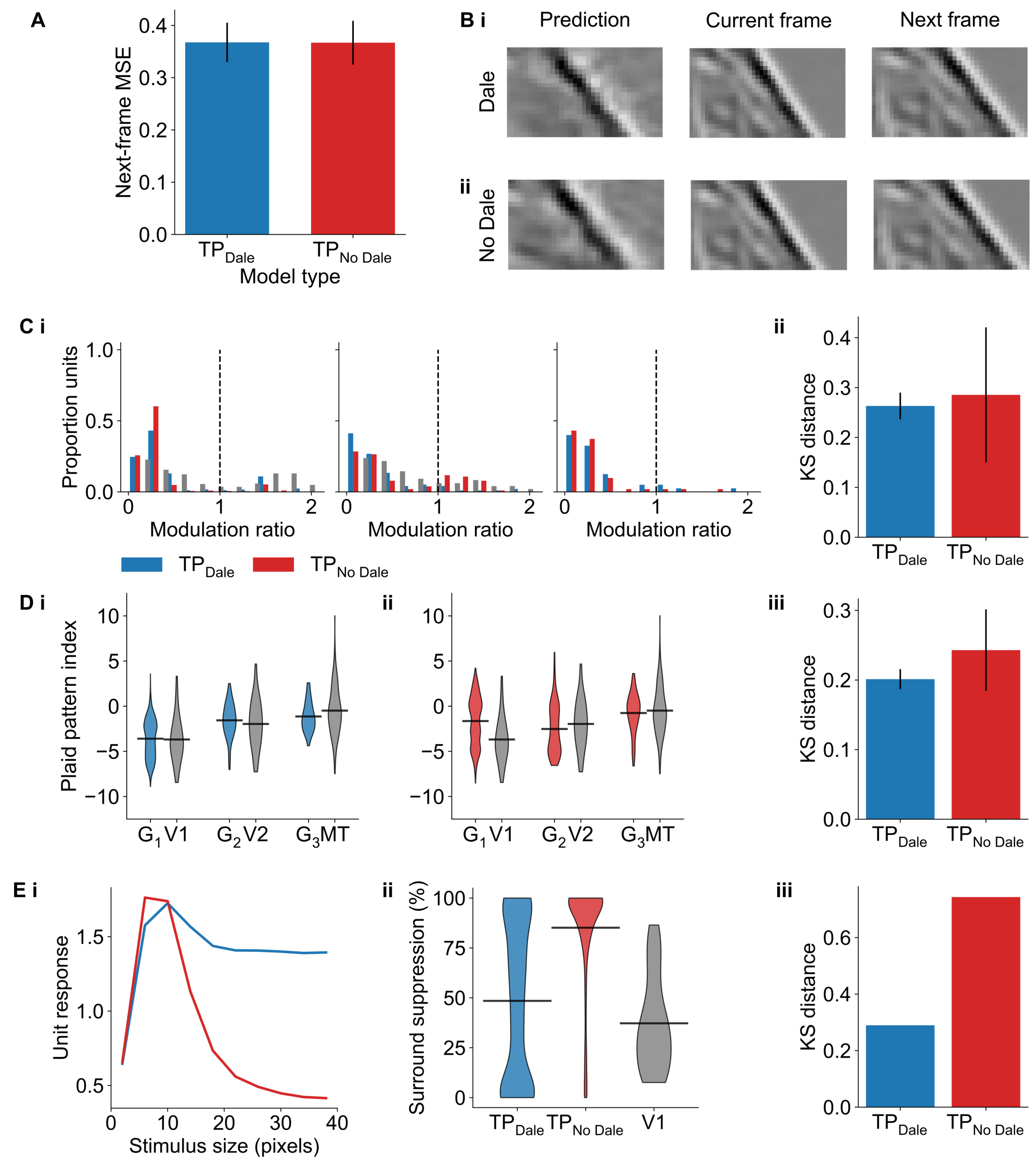

Supplement: S14 Fig — (A) Next-frame mean squared error for hierarchical temporal prediction networks that either included (TPDale) or excluded (TPNo Dale) Dale’s Law. (B) Example next-frame predictions for each network. (C-E) Distribution of model and visual cortical response properties for (C) modulation ratio, (D) plaid pattern indices and (E) surround suppression indices. These response properties more closely resembled those found in visual cortex (gray) when Dale’s Law was included in the model, as shown by the lower mean Kolmogorov-Smirnov (KS) distance for each of these measures. (TIFF) [file pcbi.1013138.s014.tiff]
