# Supplementary material for: Hierarchical recurrent temporal prediction as a model of the mammalian dorsal visual pathway
Source: PLoS Comput Biol. 2026 May 28;22(5):e1013138. doi: 10.1371/journal.pcbi.1013138 (PMC13252843; doi:10.1371/journal.pcbi.1013138)
Supplement: Attachment — Submitted filename: Response to reviewer comments final.docx [file pcbi.1013138.s016.docx]

Hierarchical recurrent temporal prediction as a model of the mammalian dorsal visual pathway (PCOMPBIOL-D-25-00956)

The reviewers’ comments are in black, our responses are in blue, and the new text added to the manuscript is included here in red. Please note that the Figs S1-14 and their captions can be found in the Supporting Information file.

**Response to reviewers' comments:**

**Reviewer #1:** In this manuscript, Klavinskis-Whiting and co-authors compare response properties of units in a model network with known properties of V1, V2, and MT neurons in macaque (and mouse). The model includes both local recurrence and inter-areal feedback and is trained to perform a hierarchical temporal prediction task on natural videos. The model recapitulates several known response properties of dorsal stream neurons, thereby offering a normative hypothesis for these properties. I enjoyed reading the paper. I think it’s great work, and the findings are important. I have a couple of suggestions for the authors to consider.

We thank the reviewer for their positive assessment.

Major suggestions
- The model representation appears complete (the number of units matches the number of pixels in a single frame). Is this important for the emerging stimulus response relations? I am asking because this seems to be the case in work that is somewhat related to this work (several papers by Bruno Olshausen on learning intermediate-level representations of form and motion from natural movies). If the model used fewer or more units than this magic number, would the overall pattern of results be similar? If possible, it would be great to explore and document this.

While we expect the relative number of hidden units to have some effect on the representation learnt, there is nothing about the model that makes completeness intrinsically important. We have now explored the model performance for a range of hidden unit numbers. Although we do find that the 800-units model performs best, we do not believe that this is an intrinsic property of the model, and this is likely to differ for other training data.

We have added the following to the Results (lines 625-637):

“We explored the effects of varying various features of the hierarchical recurrent temporal prediction model; notably the number of hidden units, the temporal statistics of the training set, the temporal offset of prediction, and the presence or absence of Dale’s law. The number of hidden units per group, within a range of 200 to 1600 hidden units, affected each network’s similarity to the visual cortex. For each network, we computed the mean KS distance between the distributions for the network and the brain across modulation ratio, plaid pattern index and surround suppression index (see Fig 5). The minimum KS distance was found for the model with 800 units, indicating that this network had the closest correspondence with the empirical distributions found in the biology (Fig S11A). Similarly, we found that the next frame prediction error reached a minimum at around 800 hidden units, while the same-frame prediction error reached a maximum at this same sized network (Fig S11B), indicating that greater similarity to the biology tended to be accompanied by improved temporal prediction performance.”

We have also added the following supplementary figure:

**Fig S11. Impact of varying the number of hidden units in the model.** Related to Fig 5. **(A)** Mean KS distance between model and neural distributions was lowest at 800 units. **(B)** As the number of hidden units increased, the model shifted from representing the current frame (same frame MSE, which increased as the number of hidden units increased) to predicting the upcoming frame (next-frame MSE, which decreased as the number of hidden units increased). Thus, a larger number of hidden units improved the network’s capacity for temporal prediction.

- One interesting property of MT neurons that is not explored by the authors is the organization of their RF in the 3-D Fourier-domain. Nishimoto and Gallant (2010) have done some beautiful empirical work on this, building on Heeger and Simoncelli’s (1998) theoretical model. While there has been less exploration of V1 neurons (a little bit in Zaharia et al., 2019 - eNeuro), this is presumably a property that emerges downstream of V1. Could you include an analysis that measures the degree to which RFs are organized along a velocity-plane?

While we agree that this would be a very interesting area to explore, this would involve coding up and fitting the switched model of Nishimoto and Gallant to our model, which would be an extensive undertaking, particularly given that the first author has now left academia. Hence, we have instead opted to discuss this phenomenon by adding the following to the Discussion (lines 728-739):

“As well as adding more biological elements to the temporal prediction model, we could compare the trained model to more diverse features of the biology, for example, frequency domain aspects. When a nonlinearly-integrated Gabor-filterbank model (inspired by earlier theoretical work, Simoncelli and Heeger, 1998) is fitted to MT neurons’ responses to naturalistic stimuli, the excitatory spectral receptive fields of the fit tend to fall on a plane within the three-dimensional spatiotemporal frequency domain, but the suppressive spectral receptive fields are found off this plane (Nishimoto and Gallant, 2011). This ensures that MT neurons are velocity tuned but are insensitive to ambiguous direction-aligned static textures. There is also evidence of somewhat similar properties in a small fraction of V1 neurons (Zaharia et al., 2019). An interesting future extension of our work would be to fit this model to the temporal prediction model so that these frequency domain properties could be examined, particularly for the higher groups in our model.”

Minor suggestions

- Figure 1, final panel: if I understand correctly, feedback connections were shuffled, but M was not retrained. Isn’t it trivial that performance decreases? Shouldn’t you show that even after retraining M, performance falls short of unshuffled R and M performance?

Apologies that we were not explicit about this. **M** is retrained every time the feedback connections are shuffled. We have now added the following to the caption for Fig. 1:

“Note that every time the feedback weights were shuffled, we retrained the output weights”

- Figure 2A: Maybe this can be quantified? How about fitting a Gabor RF and expressing the RMSE? There will likely be a systematic shift across the hierarchy. Likewise, you could use this analysis to quantify RF size and its evolution across the hierarchy.

We have performed this analysis and found that while model receptive fields in the first group could be well described as Gabors, higher groups were generally poorly fit as Gabors (Fig S4A) and showed far less spatial structure across receptive fields (Fig 2Aii-iii). Nevertheless, for that subset of group 1 units which could be modeled as Gabors (*r* >0.3)*,* we found that their receptive field size did increase across the hierarchy, as expected from the biology (Fig S4B).

We have added the following to the text to the Results (lines 310-312):

“Furthermore, consistent with our qualitative assessment in Fig 2A, the Gabor fit *r^2^* tended to decrease the higher the group and, for the units that could be well fit, the size of the fitted receptive field tended to increase (Fig S5).”

We have also added the following supplementary figure:

**Fig S5. Gabor-like receptive field properties across the model’s hierarchy.** Related to Fig 2. **(A)** Only units in the model’s first group could be modeled as Gabors, with higher groups showing a poorer fit (Gabor *r* for G_1_ vs G_2_: *t*(16.6)=7.43, *p*<0.0001). **(B)** For the subset of units that could be modeled as a Gabor (*r* > 0.3), the receptive field size – indexed by the product of the *x* and *y* Gabor standard deviation parameters – increased from group 1 to group 2 (Gabor size for G_1_ vs G_2_: *t*(7.04)=-4.22, *p*=0.004).

- Ref 22 and 34 appear to be identical. One is probably intended to be Nicole Rust’s 2006 MT paper.

You are correct that Ref 22 and 34 have been duplicated in error. Ref 34 should have been:

Movshon JA, Newsome WT. Visual Response Properties of striate cortical neurons projecting to area MT in macaque monkeys. J Neurosci. 1996;16: 7733–7741. doi:10.1523/JNEUROSCI.16-23-07733.1996

We have now updated the manuscript accordingly.

- The focus of this work is entirely on single unit response properties. Perception and prediction are of course supported by neural populations. Recent work suggests that the temporal geometry of population trajectories may play a key role here. Specifically, Hénaff et al (2019, 2021) proposed that natural movies elicit neural population trajectories that are straightened compared to retinal inputs. Niu, Savin and Simoncelli (2024, NeurIPS) explored this idea in a modeling study that is somewhat related to the current study. If the authors find this interesting, they could include a paragraph in the discussion in which they make the bridge between their current study and population level representations and perhaps suggest interesting avenues for future work to explore this space.

We agree that these are interesting and relevant studies, and we have now added the following paragraph on this topic to the Discussion (lines 825-836):

“Another related body of work is the temporal straightening hypothesis. Human psychophysics (Henaff et al., 2019), V1 neural recordings from macaques (Henaff et al., 2021) and some feedforward mechanistic models of the visual system (Henaff et al., 2019) suggest that the visual system transforms natural visual inputs to follow straighter temporal trajectories. Such a transformation has been argued to support temporal prediction in natural environments (Henaff et al., 2019; 2021). A feedforward visual processing model optimized for temporal straightening was found to increase recognition robustness more than a similar invariance-optimized model (Niu et al., 2024). Conversely, visual processing models adversarially optimized for robustness were found to increase temporal straightening, something not necessarily found in other visual processing models (Harrington et al., 2023). It would be interesting to examine in future work the effect of the hierarchical temporal prediction objective on both recognition robustness and temporal straightening in our recurrent networks.”

**Reviewer #2:** Klavinskis-Whiting et al. propose an artificial neural network (ANN) model of the dorsal visual stream in the brain. The model relies primarily on unsupervised hierarchical predictive training and incorporates architectural components such as feedback connections. The authors provide evidence that these elements are necessary for capturing certain functional properties of the dorsal pathway. In particular, they demonstrate their model's capability in reproducing frequency tuning, surround suppression, and pattern motion selectivity across dorsal areas of the monkey visual system. Additionally, through representational similarity analysis, they benchmark their model against several baseline models in terms of similarity with the mouse visual cortex. The paper is very well written and presents a compelling computational model of the dorsal visual stream. I am generally very supportive of the publication of this paper, but I do have some comments and questions that I detail below.

We thank the reviewer for their positive assessment.

Major Comments:

- Could the results section include more detailed explanations of the model architecture and modules? The G modules specifically remain somewhat obscure without consulting the methods section, and providing key architectural insights in the results would improve accessibility.

We now provide a more detailed explanation of the model in the Results on pgs. 4-5:

“More explicitly, the network is essentially a single-hidden layer recurrent neural network (Elman net) with various constraints applied to provide hierarchical structure. All the units of the model are rate-based. At time *t*, the hidden unit activity vector **h**[*t*] of the network is given by:

$$\mathbf{h}\left[ t \right]=f(\mathbf{Rh}\left[ t-1 \right]+\mathbf{Wu}\left[ t \right]+\mathbf{b})$$

(1)

where **R** is the recurrent weight matrix, **W** is the input weight matrix, **b** is the input bias vector, **u**[*t*] is the input vector, and f(•) is a rectified linear unit (ReLU) nonlinearity. The input vector consists of a frame at time t from a movie of natural scenes of 40 timesteps in duration, with the 20x40 pixel frame flattened into a length 800 vector. This equation, with constraints that are outlined below, comprises our model of the dorsal pathway, which is compared with neural data.

The output vector **y**[*t*] of the network, which is a prediction of future input and activity, is given by:

$$\mathbf{y}\left[ t \right]=\mathbf{Mh}\left[ t \right]+\mathbf{c}$$

(2)

where **M** is the output weight matrix and **c** is the output bias vector.

Hierarchy is imposed on the network in three ways. First, the hidden unit vector is divided into three equally sized groups of 800 units each, **h** = (**h**^1^, **h**^2^, **h**^3^), where the superscript denotes group number. Hierarchy is encouraged by having no connections between group 1 and group 3 - that is by fixing the corresponding regions of the **R** matrix to zero. The output vector **y** is also divided into three groups, **y** = (**y**^1^, **y**^2^, **y**^3^), of corresponding lengths *K*^1^=*K*^2^=*K*^3^=800 and the **M** matrix is fixed to zero except for the weights from **h**^1^ to **y**^1^, **h**^2^ to **y**^2^ and **h**^3^ to **y**^3^. Second, the input matrix **W** only connects to group one, that is all weights are fixed at zero except those going into group 1. Finally, the most important source of hierarchy comes from the loss function used to train the network. The loss *L* was given by:

$${\begin{aligned} L=\lambda Z+ \\ \left\langle\frac{1-\beta}{K^{1}}\left\| \mathbf{y}^{1}\left[ t \right]-\mathbf{u}\left[ t+1 \right] \right\|_{2}^{2}+\frac{\beta}{K^{2}}\left\| \mathbf{y}^{2}\left[ t \right]-\mathbf{h}^{1}[t+1] \right\|_{2}^{2}+\frac{\beta}{K^{2}}\left\| \mathbf{y}^{3}\left[ t \right]-\mathbf{h}^{2}[t+1] \right\|_{2}^{2} \right\rangle\end{aligned}}_{n,t}$$

(3)

Here, group 1 predicts the input, group 2 output predicts group 1 hidden unit activity, and group 3 predicts group 2 hidden unit activity. The L2 norm is given by ||•||_2_ and the expectation over all clips and time given by <•>*_n,t_*. The clip subscript *n* is left off the vectors for simplicity. The regularizer *Z* is the sum of the absolute value of all the weights in the network (**W**, **R** and **M**), and can be seen as a constraint on connectivity, and is scaled by *λ*. Crucially, the hyperparameter *β* governs the relative importance in the loss function of predicting the input versus higher groups predicting lower groups.

An additional constraint is that 20% of the hidden units in each group were constrained to be inhibitory; that is, the weights from them in the recurrent connectivity matrix (**R**) were constrained to be negative, and they only make intra-group connections. All other hidden units were constrained to be excitatory; that is, the weights from them in the **R** matrix were constrained to be positive. For a visual depiction of the constraints on the network see Fig S1.

We trained the network by minimizing the loss function with respect to the weights (**W**, **R**, **M**) and biases (**b**, **c**) using the Adam backpropagation algorithm, for a large dataset of movies of natural scenes. We used backpropagation as our focus was on whether the functional organization of the dorsal pathway is consistent with being optimized for hierarchical recurrent temporal prediction, and not the exact learning mechanism by which this is achieved. The number of active hidden units in the trained network, as measured by the response to a visual noise stimulus, was 800 units in group 1, 97 units in group 2, and 42 units in group 3. Note that this is consistent with primary visual cortex being typically larger and having greater neuronal density than higher visual areas. We take the active hidden units of the trained network to correspond to neurons in the dorsal pathway.”

We have also added the following supplementary figure:

**Fig S1. Visual illustrations of the groupings and constraints applied to the weight matrices and activity vectors of an Elman network to produce the model.** Related to Fig 1. The squares and broad vertical rectangles illustrate the weight matrices. The shade (dark/mid/light) indicates what group (G_1_/G_2_/G_3_) the weights in that region of the matrix project from. The color (white/red/blue/gray) indicates whether those weights are constrained to be zero (white), positive (red), negative (blue) or unconstrained (gray). The narrow vertical rectangles illustrate the vectors, with shade indicating grouping and color indicating whether they are excitatory units (red), inhibitory units (blue) or unconstrained output units (gray). Vectors have been widened slightly for visualization purposes only. Bias vectors have been left off for simplicity. **(A)** An illustration of the equation for the hidden unit activity (Equation 1). This shows the input vector, **u**[*t*], the input weight matrix, **W**, the hidden unit vector, **h**[*t*], and the recurrent weight matrix, **R**. Note the inhibitory (blue) and excitatory (red) units of the hidden unit vector and their corresponding constrained weights in the recurrent matrix. **(B)** An illustration of the equation for the network output (Equation 2). This shows the output vector, **y**[t], and output weight matrix, **M**, along with the hidden unit vector to which the output weights are applied. **(C)** An illustration of the loss function (Equation 3), using the input vector and groups (**h**^2^[*t*], **h**^2^[*t*], **y**^1^[t], **y**^2^[t], **y**^3^[t]) from the hidden and output vectors.

- Prior works on using ANNs in modeling the dorsal pathway is lacking, especially those that have demonstrated similar neural phenomena (particularly pattern motion tuning) using different architectures and/or loss functions (DorsalNet: Mineault et al., NeurIPS, 2021, MotionNet: Rideaux and Welchman, Journal of Neuroscience, 2020). A comparison with existing models could strengthen the contribution.

We agree that this would strengthen the paper, and we have now added the following paragraph on this topic to the Discussion (lines 810-823):

“Two further studies examined whether properties of the dorsal pathway could be explained by networks optimized to estimate certain gross velocity measures from visual input. These measures were either the overall direction and speed of the whole moving scene (Rideaux and Welchman, 2020) or head and body motion of agents in simulated environments (Mineault et al., 2021). The first study showed that their network could explain neural and perceptual biases for particular motion directions, the interrelation between speed and direction preferences in MT neurons, and certain motion illusions. The second study showed that their network to a degree captured in a hierarchical manner the responses of neurons along the visual pathway to naturalistic stimuli. An interesting extension of this would be to develop a multisensory hierarchical recurrent temporal prediction model that combines temporal prediction of these gross velocity measures, as might be provided by vestibular inputs, with the prediction of spatially detailed future visual input that we use here. We suspect that both objectives are important, with different properties of the dorsal pathway being more relevant to one or the other objective.”

- The explanations surrounding Bakhtiari et al. citation contains some inaccuracies. I am very familiar with that work, so I know that the ResNet backbones in that study were actually trained from scratch using contrastive self-supervised learning -- not pre-trained architectures. Also, in general, I don’t agree with the authors that previous contrastive models mainly relied on pretrained ResNets.

We thank the reviewer for this correction and have deleted the sentence in question.

- Are the baseline models equalized in terms of parameter count? Removing components like feedback connections would decrease parameter count, which may affect the interpretation of results. The authors might consider providing comparisons with parameter-equalized models, or alternatively, could clearly report the parameter counts for each model and discuss why differences in parameter count are not expected to influence the results.

To clarify on this matter, we have now added the following paragraph to the Methods (lines 1005-1020):

“The recurrent autoencoder and recurrent temporal prediction networks had exactly the same parameter counts (13.4 million trainable parameters) versus a slightly lower number for the feedforward temporal prediction network (11.5 million trainable parameters). This represents a small (14%) reduction in parameters, largely due to the absence of feedback. While we could have produced a feedforward model that equalized the number of parameters, we this would have come at the expense of increasing the number of hidden units in the feedforward model. Thus, there is an inevitable trade-off between equalizing the number of total parameters versus the total number of hidden units across networks.

In terms of the experiments where we ablated feedback from a model that initially included feedback connections, this necessarily reduced the total parameter count. However, feedback was removed via ablation, analogous to the experimental data, where reducing feedback in the actual brain involves reducing the number of connections. Thus, we believe this ablation approach is the most appropriate for the questions we were trying to answer about the biology.”

- For the memory experiments, would it be possible to train a decoder on frame 0 and test on n-back frames? This approach would provide a more precise measure of information retention over time.

We have performed the analysis as suggested, and added the following sentence to the Results (lines 260-261):

“We retrained the decoder for each n-back condition since a fixed decoder is insufficient (Fig S3).”

We have also added the following supplementary Figure:

**Fig S3. MNIST decoder accuracy across n-back frames when training on a 0-back decoder for all n-back conditions.** Related to Fig 1. Here we trained a 0-back decoder and tested this fixed decoder for each n-back condition. We found that the decoder – in both the full and no feedback conditions, as well the shuffled feedback condition – was unable to generalize from the 0-back condition and performed at chance. Given the performance of the model in Fig 1J-K when trained and tested on the same n-back condition (with separate train and test splits), it is unlikely that these fixed 0-back decoder results reflect an absence of information in the network over time. Rather, this suggests that using a fixed decoder is not sufficient and that it needs to be re-trained for each n-back condition to extract the information, as we do in Fig 1J-K. **(A)** MNIST accuracy declines to chance at decoding from the n-back condition if a fixed 0-back decoder is used, for both the full and no feedback models. **(B)** MNIST accuracy at 5-back performs at chance as the percentage of feedback is shuffled for a fixed 0-back decoder. In both cases, chance performance indicates that the decoder is unable to generalize from the 0-back condition.

- The representational similarity analysis with the mouse visual system is very informative. Could the authors better justify the choice of mouse visual areas to represent the dorsal pathway? There is some disagreement in the literature regarding which areas should be included in specific visual pathways in mice (e.g., Yu et al., Current Biology, 2022, Sit and Goard, Nature Communication, 2020) as the distinction isn't as clearly established as in primates. Additionally, the hierarchical organization among these areas remains unclear, making direct comparisons with macaque V1 and MT potentially problematic. It might be worth considering a broader examination of mouse visual areas (beyond LM and RL) independently. Notably, pattern neurons have been reported even in area LM (Matteucci et al., Science Advances, 2023), which may blur the functional boundaries between areas in the mouse visual system.

We agree with the reviewer that these complications should be made more apparent. Hence, we have updated the relevant paragraph in the Results (paragraph beginning line 567) to read:

“In terms of brain regions, we considered mouse V1 as well as two early regions of the ventral (rostrolateral visual cortex) and dorsal (lateromedial visual cortex) visual streams. In the primate visual system, the dorsal- and ventral-streams are characterized by their relative selectivity to motion and form, respectively (Mishkin et al., 1983). The mouse visual system similarly may feature dorsal- and ventral-like streams, which have been suggested to respectively contain the rostolateral and lateromedial cortical regions (Wang et al., 2011, 2012; Yu et al. , 2022). However, the distinction between these streams is less well characterized than in macaque monkeys and perhaps less distinct. For example, one study found that while the rostrolateral region showed strong sensitivity to motion in natural scenes, it was only moderately sensitive to motion in random dot kinematograms (Sit and Goard, 2020). Conversely, a study in anesthetized rats (also in the family Muridae) showed more pattern-motion-selective cells in lateromedial than rostrolateral cortex (Matteucci et al., 2023), the opposite of findings in mice where rostrolateral cortex was found to have more (Juavinett and Callaway 2015). Nonetheless, the majority of studies in mice suggest rostrolateral visual cortex is more sensitive to motion than lateromedial visual cortex in mice, broadly consistent with the functional distinction between cortical areas in the primate visual system (Sit and Goard, 2020). Accordingly, mice provide an opportunity to investigate how the motion tuning of the temporal prediction model relates to the model-brain alignment in the stimulus representations across these three visual areas.”

We agree that a broader comparison to mouse visual areas would be valuable, but we will reserve this for a future study.

- How much might the specific training video content have influenced the results? Testing with alternative video datasets could help establish whether the findings are driven by the objective function and architecture rather than training data characteristics. This consideration is particularly important given that with unsupervised predictive training, the visual features that form the model's representations are expected to be influenced by the statistical properties of the training data. Could the authors comment on the potential impact of training data selection on their findings? It would also be helpful to include representative frames from the training videos in the supplementary materials.

In an earlier part of the study, we trained a series of networks using an entirely separate dataset and found that the networks similarly learned Gabor-like receptive fields and could well describe the representational characteristics of the mouse visual cortex. Thus, we believe that the results described in the manuscript are fairly robust to the specific dataset used for training so long as it is sufficiently rich and naturalistic.

On this point have added the following paragraph to the Results (lines 639-648):

“We expect that the statistics of the training dataset will have an impact on the representations learned by the network. Indeed, at an extreme, we found that training the network on the same dataset but removing temporal contiguities by shuffling the order of frames within a clip, prevents the network from learning useful representations. For example, compare the receptive fields learned by the network where the frames are shuffled (Figure S12Ai) with those learned by the standard temporally contiguous dataset, which show a clear Gabor-like structure (Figure S12Aii). Nevertheless, the network was trained on a large diverse dataset of naturalistic stimuli (Figure S12B; example frames) and we believe that any sufficiently rich, naturalistic dataset like the one used in this study would result in broadly similar network properties.”

We have also added the following supplementary figure:

**Fig S12. Impact of network training dataset and exemplar frames.** Related to Fig 5. **(A)** A network trained on the same dataset but with temporal contiguities removed by shuffling the frame order fails to produce coherent receptive fields (i) Conversely, the standard temporally-contiguous dataset produces receptive fields with a clear Gabor-like structure (ii). **(B)** Example frames from the dataset used for model training.

- Regarding the feedback effects on plaid responses, is it clear that neurons actually convert to component-selective responses? The removal of feedback seems to disrupt tuning properties more generally, and the pattern index may not adequately capture these effects (e.g., compare panels 3Bi vs 3Fii).

While we agree with the reviewer that tuning properties certainly do change with the removal of feedback in the network – as in the biology when feedback is removed (Schmidt et al., 2011) – we note that the reduction in plaid tuning index is relatively small and thus does not completely abolish pattern- (or indeed component-) selective responses. Indeed, comparing the preferred direction tuning of direction selective units in the network, we find only a 6.6° change in tuning preference after abolishing feedback (*t*(579)=-4.4, *p*<0.001), indicating that tuning to the underlying grating stimuli remains relatively consistent. Moreover, while the example provided in Figure 3F shows a more dramatic change in plaid tuning, there are many examples of neurons with more subtle changes that show clear component responses when feedback is abolished (Figure S7).

To this end we have added the following to the Results (lines 412-416):

“Indeed, suppressing feedback from the middle suprasylvian gyrus has been shown to reduce pattern-like responses in early visual cortex of the cat [33]. In line with these experimental data, abolishing feedback in the model resulted in a significantly lower mean plaid pattern index in G_1_ (t-test, *t*(1519)=5.33, *p*<0.0001; Fig 3E), which at the level of individual model units often resulted in a change from a plaid-like response (Fig 3Fi) to one that more closely resembled a component-like response (Fig 3Fii, Fig S7). Furthermore, we find that G_1_ units show a mean magnitude change in direction tuning to simple moving gratings of only 6.6° after we ablated feedback (*t*(579)= -4.4, *p*<0.001), indicating that tuning to the underlying grating stimuli remained relatively consistent. This is consistent with the impact of the feedback ablation on the plaid pattern index not simply being a consequence of a general disruption of direction tuning.”

We have also added the following supplementary figure:

**Fig S7. Example model units (i-iii) showing changes in plaid tuning after the removal of feedback connections.** Related to Fig 3.

Minor comments:

- Why not include spatial and temporal frequency tuning comparisons in the model evaluations? This seems particularly relevant given the proposed model's difficulty with spatial frequency patterns.

We have now included the following on this point in the Results (lines 547-553):

“We also performed a comparison of spatial and temporal frequency tuning across model types. The relative spatial frequency tuning across model groups was relatively consistent across the different models (Fig S9Ai-Ci), showing a U-shaped profile with spatial frequency tuning highest in the first and last groups. In contrast, temporal frequency tuning was more similar to the data for the hierarchical temporal prediction model (Fig S9Aii) than for either the feedforward model (Fig S9Bii) or the autoencoder model (Fig S9Cii).”

We have added the following supplementary figure:

**Fig S9. Spatial and temporal frequency tuning across model types.** Related to Fig. 5. Spatial frequency tuning (i) and temporal frequency tuning (ii) across the **(A)** hierarchical recurrent temporal prediction model, **(B)** feedforward temporal prediction model and **(C)** autoencoder model compared with data from the macaque visual cortex.

- Could the authors clarify how cycles per degree are transformed to cycles per pixel in the tuning analyses? This would help with proper comparison to existing data.

To clarify on this point, we have added the following to the Methods (lines 1037-1041):

“Because the input visual stimuli used to train and analyze the network have no fixed reference point, we do not think there is a reliable way of converting between pixel space and degrees in visual space. Thus, we take the comparison between experimental measurements in cycles/degree and model results in cycles/pixel as best understood on a relative, rather than absolute, basis.”

- Can the authors explain what happened when feedback was removed in the autoencoder model? Why did all responses drop to near zero?

We have now looked into this further and confirmed this effect, indicating that in this context for the autoencoder the feedback has a disinhibitory effect. We have now added the following to the Results (lines 531-535):

“Indeed, when feedback is abolished in the autoencoder model, the units’ responses became much weaker but tended to remain above zero (mean unit response of 0.11). Thus, in this context, feedback in the autoencoder model appears to have a disinhibiting effect on the network compared with the more suppressive effect in the hierarchical temporal prediction model.”

**Reviewer #3:** ======. Summary ======

The authors present a biologically inspired deep network in which each layer is trained to predict the future activity of the layer below. They compare this hierarchical predictive model to two alternatives: (i) a model without feedback connections, and (ii) a model trained to predict the current rather than future input (i.e., a deep autoencoder). They find that the predictive model captures various features of visual cortical responses—such as motion tuning, surround suppression, and responses to overlapping plaids—more accurately than the alternative models.

=======Strengths ======

* The research question is timely and of clear relevance to the field.
* The proposed approach is well-motivated and the methodology appears generally sound.
* The conclusion—that the predictive model better accounts for a range of visual neuronal properties than the controls—is supported by the simulation results.

Overall, I consider this to be a strong paper, and I recommend publication pending minor revisions. I do, however, have several questions that I believe the authors should address:

We thank the reviewer for their positive assessment.

=======Major Questions=======

1. **Interpretability of the Results**

The results are primarily correlational: the predictive model shows better alignment with biological data, but the underlying reasons remain unclear. Can the authors provide some theoretical insight into why the predictive model better accounts for effects like surround suppression, as compared to the autoencoder trained on present input? This is important for interpreting whether the observed behaviors are intrinsic to hierarchical predictive coding architectures, or whether they might result from implementation-specific choices (e.g., the choice of loss function, nonlinearity, enforcement of Dale’s law, etc.). While this is a challenging question, some analysis or discussion along these lines would significantly strengthen the paper.

We have now added two paragraphs on this point to the Discussion (lines 763-785):

“The autoencoder used exactly the same architecture as the temporal prediction networks but was substantially worse at explaining the neural responses. This suggests that it is the temporal prediction objective that is particularly critical for reproducing the biological data. However, it is still the case that appropriate architecture, constraints and training data, such as hierarchy and recurrency (Fig 5), Dale’s law (Fig S8), appropriate prediction offset (Fig S9), and temporally-structured stimuli (Fig S12), can help produce brain-like properties. Similarly, we have shown in earlier work that such factors are important for simple feedforward (Singer et al., 2018; Trinh et al., 2023), hierarchical feedforward (Singer et al., 2023), and non-hierarchical recurrent temporal prediction networks (Klavinskis-Whiting et al., 2025). Our previous studies also explored the importance of other constraints, such as connectivity strength (L1 regularization strength) and noise levels. Thus, these further constraints will also likely be relevant to the hierarchical recurrent temporal prediction model.

For temporal prediction models, these constraints are often tuned to optimize prediction, rather than simply to make the model look like the brain. Hence, temporal prediction has an independent criterion for tuning hyperparameters, something the autoencoder lacks. In contrast, an autoencoder with exactly these same constraints can only poorly replicate brain-like properties, even when these constraints, such as the L1 regularization, are tuned to make the network more like the brain rather than an independent criterion. Furthermore, our earlier work has also shown that temporal prediction can account for various receptive field properties better than sparse coding, predictive coding, slowness, inpainting or denoising models. Together, this suggests that temporal prediction represents a good normative principle for sensory processing in the brain.”

2. **Interpolation Between Architectures**

Can the authors explore a continuous interpolation between the predictive and autoencoding models—for example, by gradually shifting the prediction target from the present to various future time points (in the multi-frame setting)? Such an approach might help clarify the functional differences between the two models.

We have now explored this for the models, and added the following paragraph to the Results (lines 650-671):

“To better understand the specific role of temporal prediction in the network model, we interpolated between an autoencoder and temporal prediction model by shifting the temporal offset of the frame target from 0 to 42 ms into the future (see Methods). For each temporal offset, we computed an aggregate score as the mean Kolmogorov-Smirnov (KS) distance for each of the measures described in Fig 5 – namely, the modulation ratio, plaid pattern index and surround suppression index (Fig S13A). Overall, we found that the 0 ms temporal offset (autoencoder) model had the highest score – and was therefore most different from the comparison neural data – whereas the model trained to predict a target 42 ms into the future was most similar to the neural data.

We next fitted each group 1 unit with either a Gabor or Difference of Gaussians (DoG) function (Fig S13B). As the temporal offset increased, group one units became more Gabor-like, and less DoG-like. Finally, we investigated the representational similarity of each network to the mouse visual cortex as we increased the temporal offset (Fig S13C). Overall, we found that models with a temporal offset greater than 8 ms best captured the representational geometry of the mouse visual cortex across all three areas surveyed. Interestingly, we found that for both V1 and lateromedial visual cortex (part of the mouse ventral visual cortex), further increases in representational similarity saturated at 17 ms into the future. Conversely, we found that for rostrolateral visual cortex (part of the mouse dorsal visual stream), there was a distinct benefit to predicting at 25 ms into the future, implying that this region of the dorsal stream is more sensitive to the precise temporal horizon of prediction.”

We have added the following supplementary figure:

**Fig S13. Role of the temporal prediction offset in network properties.** Related to Fig 5. **(A)** The mean KS distance between the model and brain distributions of modulation ratios, plaid pattern indices and surround suppression indices. A lower score indicates a more brain-like distribution in the model, with the autoencoder having the least brain-like distribution. **(B)** The distribution of Gabor-like versus difference-of-Gaussian-like units in group 1 across temporal offsets, with the highest proportion of Gabor-like units found when predicting the next frame 42 ms into the future. **(C)** Representational similarity between the model and different regions of mouse visual cortex across temporal offsets.

3. **Stimulus Choice in the Multi-frame Setting**

In the multi-frame experiments, the authors restrict themselves to a simplified moving bar stimulus. Why not evaluate performance on naturalistic movie stimuli as well? This would seem a natural extension, and might yield more informative or generalizable insights.

We performed this analysis for naturalistic movie stimuli but did not find improved multi-frame prediction for non-zero beta.

Minor comments/Questions

- How does the comparison between predictive network and biological detail depend on parameters of objective function such as beta (which determines relative weighting to each layer in the network) and lambda (the L1 norm)? If effort was made to tune these, so as to best fit biological data, was comparable effort made for the controls?

The reviewer is correct, we did tune these parameters for both models. We have now added the following sentence clarifying this point to the Methods (lines 996-999):

“For the temporal prediction model and the control model, some exploration of hyperparameters λ and β was performed to find those values that produced biological realistic receptive fields, with the temporal prediction also having the additional independent criterion that prediction error remained small.”

See also the new Discussion paragraph in response to major point 1.

- I was confused by the role of beta. The authors claim in line 143 that "A higher beta value indicates a greater weighting applied to higher groups,". Doesn't this imply that small beta corresponds to the network being more 'feed-forward' since lower layers will receive relatively less input from higher groups? This would seem to contrast their statements in lines 148-151.

Beta determines how much the loss function weights predicting the input vs higher groups predicting lower groups. A lower beta would favor predicting the input, whereas a higher beta would favor higher groups predicting lower groups (whose activity is a non-linear transformation of the input). A small beta would not necessarily be more feedforward, however, as local recurrent connections could still be useful in predicting the input. Likewise, feedback connections from higher groups might still be useful by providing additional processing machinery and delays for input prediction. We speculate that a small beta of the appropriate size might encourage group 2 and group 3 to represent predictive features of the stimulus in latent and more nonlinearly transformed spaces, which perhaps enables better input prediction on novel stimuli. If the value of beta is too low, the higher groups would still provide processing machinery, but it may be less useful for input prediction of novel stimuli. If the value of beta is too high, the higher groups would bias the network away from the direct input prediction of group 1, perhaps by preferably driving group 1 activity to be predictable rather than predictive of the input.

The new text at the start of the Results section on pgs. 4-5 provides a more detailed introduction to the hierarchical recurrent temporal prediction model, which includes the equation for the loss function and also now explicitly states (lines 165-166):

“Crucially, the hyperparameter *β* governs the relative importance in the loss function of predicting the input versus higher groups predicting lower groups.”

We hope that this provides clarification on the role of beta. For more details, please see our response to the first major comment of Reviewer #2.

- In the initial presentation of the model (lines 117-141) I felt that there should have been more description of model details. For example, without going to the methods section we don't know if it is a rate or spiking model, what is the non-linearity, how the learning works etc. I appreciate that these details are in the methods, but since this is at the end, I think the results should have more details, otherwise the reader has to constantly refer back and forward to the methods, which is difficult.

We agree and Reviewer #2 made the same point. We have now added a more detailed introduction to the hierarchical recurrent temporal prediction model at the start of the Results section. Here we make clear that it is a rate model, that the non-linearity is ReLU, and how the learning works, among many other details. The new text is included above as part of our response to the first major comment of Reviewer #2.

- I think there could be more description about what are the bottlenecks/constraints in the model, for example the L1 norm. Without these, there is nothing to stop e.g. the 2nd layer just reproducing the activity of the 1st layer identically (which allows best prediction since no information is lost). Thus, given the key role of constraints/costs I think they're relatively glossed over in the main paper.

Even without the L1 constraints, we do not think the 2^nd^ group would simply reproduce the activity of the first layer identically. This is because the activity of the 1^st^ group is a lossy nonlinear transformation of the input due to the ReLU and loss of non-predictive information. Thus, the 2^nd^ group, in trying to predict the activity of the 1st group, would be focused on capturing patterns across these non-linear features that predict their future presence, which is a substantial abstraction from the input and would necessitate a different representation in the 2^nd^ group.

The expanded Results section now provides a more detailed introduction to the hierarchical recurrent temporal prediction model. In this text, we explicitly lay out all the constraints. We have also added a visual depiction of these constraints in our new supplemental figure S1.

- The authors emphasize that the network satisfies certain biological constraints, such as Dale’s law. What is the impact of this constraint on the results? Would the model's behavior or predictive performance change significantly if this constraint were relaxed?

We have now explored this property of the model, and added the following paragraph to the Results (lines 673-682):

“To probe the role of Dale’s law in the network’s learned properties, we compared two networks trained for hierarchical temporal prediction, differing only in the inclusion of Dale’s law. Overall, including or excluding Dale’s Law had almost no impact on next-frame prediction performance (Fig S14A), and, visually, the two networks’ next frame predictions were very similar (Fig S14B). Nevertheless, in terms of the networks’ learned properties, we found that the model with Dale’s law was more like the neural data, implying that this added biological detail produced a more brain-like network. In particular, we found that the distributions of modulation ratios (Fig S14C), plaid pattern indices (Fig S14D) and surround suppression indices (Fig S14E) were more like those reported in the visual cortex for the model that included Dale’s law.”

We have added the following supplementary figure:

**Fig S14. Comparison of network properties when including or excluding Dale’s Law.** Related to Fig 5. **(A)** Next-frame mean squared error for both networks. **(B)** Example next-frame predictions for each network. **(C-E)** Distribution of model and visual cortical properties for (C) modulation ratio, (D) plaid pattern indices, and (E) surround suppression indices.

======= Minor Comments and Questions=====

The following points appear to be a copy of the previous section, so for our responses please see above.

1. **Sensitivity to Hyperparameters**

How does the comparison between the predictive network and biological data depend on the parameters of the objective function, such as *β* (which controls the relative weighting of each layer) and *λ* (the L1 norm coefficient)? If the authors made efforts to tune these parameters for the predictive model to best fit biological data, was a comparable effort made for the control models?

2. **Clarification on Role of β**

The role of *β* is somewhat unclear. On line 143, the authors state that “a higher beta value indicates a greater weighting applied to higher groups.” This would seem to imply that smaller *β* values make the network more feedforward, as lower layers receive proportionally less influence from higher ones. However, this appears to contrast with the interpretation in lines 148–151. Could the authors clarify this apparent contradiction?

3. **Lack of Model Detail in Initial Presentation**

In the initial description of the model (lines 117–141), important architectural and implementation details are missing. For example, it is not clear whether the model is a rate-based or spiking network, what the nonlinearity is, or how learning is implemented. While these details are included later in the Methods section, the lack of information in the main text makes the Results difficult to follow without frequent back-and-forth reference to the Methods. Including a brief summary of key model features earlier in the text would improve clarity.

4. **Underemphasis of Architectural Constraints**

The paper would benefit from a more explicit discussion of the architectural constraints that limit the model’s representational capacity—such as the L1 sparsity penalty. Without such constraints, for example, the second layer could in principle replicate the activity of the first layer exactly, achieving perfect prediction with no information loss. Since the model’s behavior depends critically on these constraints, it would be helpful if the main text gave them greater emphasis and clarified their functional role.

5. **Impact of Dale’s Law**

The authors highlight that the model enforces Dale’s law. However, it remains unclear what effect this constraint has on the results. Would the behavior or predictive performance of the model change meaningfully if Dale’s law were removed? Some comment or ablation analysis would be valuable here.
