# Supplementary material for: Hierarchical recurrent temporal prediction as a model of the mammalian dorsal visual pathway
Source: PLoS Comput Biol. 2026 May 28;22(5):e1013138. doi: 10.1371/journal.pcbi.1013138 (PMC13252843; doi:10.1371/journal.pcbi.1013138)
Supplement: Attachment — Submitted filename: response to reviewers final.docx [file pcbi.1013138.s017.docx]

PCOMPBIOL-D-25-00956R1
Hierarchical recurrent temporal prediction as a model of the mammalian dorsal visual pathway
PLOS Computational Biology

**Response to reviewers' comments**

*Our responses to the reviewers’ comments are in blue and the new text is in red*

**Reviewer #1**

The authors have addressed my concerns. It is a beautiful piece of work.

We would like to thank the reviewer for their comments, which we feel have greatly improved the paper.

**Reviewer #2**

I thank the authors for their thorough revisions. I have a remaining question regarding the role of feedback in pattern motion selectivity.

Specifically, there appears to be a discrepancy between the results in Figure 3F and those in the new Supplementary Figure S7. In Figure 3F, the tuning curves become notably noisy following feedback removal, suggesting a general degradation of motion selectivity rather than a clear shift toward component selectivity. In contrast, the tuning curves in Figure S7 align much more closely with the paper’s hypothesis. Could the authors clarify why these two sets of results differ? Furthermore, given that Figure S7 provides a clearer demonstration of the proposed mechanism, I would encourage the authors to explain why Figure 3F was selected for the main text instead.

We described the removal of feedback as resulting in “a change from a pattern-like to a component-like response when feedback is abolished”, but we agree with the reviewer that the previous version of Figure 3Fii (no feedback) showed a rather noisy (albeit not entirely untuned) unit. And we further agree that Figure S7 includes other example units that show a much clearer transition from pattern-like to component-like responses following the abolition of feedback. As a result, it may be fairer to describe the abolition in feedback as resulting in a mix of component-tuned responses and units with degraded motion tuning.

To make this clearer, we have switched the unit previously shown in Figure 3F with the unit in Figure S7i, while amending the text to highlight that the abolition of feedback has a more heterogenous impact on motion tuning (line 378-383):

“In line with these experimental data, abolishing feedback in the model resulted in a significantly lower mean plaid pattern index in G1 (t-test, *t*(1519)=5.33, *p*<0.0001; Fig 3E). At the level of individual model units, this often resulted in a change from a plaid-like response (Fig 3Fi) to one that more closely resembled a component-like response (Fig 3Fii, Fig S7i-ii), though other units had more varied responses and appeared to show more generally degraded motion tuning following the abolition of feedback (Fig S7iii).”

**Reviewer #3**

I commend the authors on the hard work they have put in to addressing my, and the other reviewers' comment. I recommend for publication.

I just have a couple of tiny points regarding their answers to my comments:

(1) For the multi-frame setting the authors responded that they found no improved multi-frame prediction for non-zero beta.

That is correct. Although we found a small improvement using a non-zero beta for the moving bar stimuli (as shown in Figure 1H and accompanying text), we found no improvement for the naturalistic stimuli.

(2) Regarding parameter tuning the authors have added "“For the temporal prediction model and the control model, some exploration of hyperparameters λ and β was performed to find those values that produced biological realistic receptive fields, with the temporal prediction also having the additional independent criterion that prediction error remained small.” Could they be more explicit about what objective criteria were used to choose their parameters in each case (and including the controls)?

We apologize that this wasn’t expressed more clearly. More specifically, for the temporal prediction models, we varied the λ hyperparameter across a range of values and computed the next-frame MSE on a held-out test-set. We found that the optimal λ value produced less spatially distinct receptive fields than found in the biology, so we chose a nearby higher value that gave more spatially defined receptive fields while remaining close to the normative-driven optimum. For the autoencoder model, as there is a less well-defined objective value for the hyperparameters (the reconstruction error almost always improves for a less ‘constrained’ network), we chose the λ hyperparameters that gave the most spatially well-defined receptive fields and which best represented what is found in the visual cortex. The hyperparameter β was explored during model development but less systematically than λ, and the value chosen (β = 0.1) was one that gave visual-system-like properties for the different model types and good out-of-distribution prediction for moving bars.

We have rewritten this section of the Methods (lines 924-938):

“The temporal prediction model and its variants were all implemented in PyTorch, with gradient descent performed using the ADAM optimizer set at a learning rate of α = 10^-4^. The hyperparameter β was set at 0.1, as this value gave cortex-like properties for the different model types and good out-of-distribution prediction for moving bars. More systematic exploration of hyperparameter λ was performed, which was set as 10^-6^, close to the global minimum that minimized the mean squared error for next frame prediction on the validation dataset while producing the most biologically realistic receptive fields. For the temporal prediction model, we found that the optimal λ value produced receptive fields that were similar to but not as spatially distinct as those found in the cortex, so we chose a nearby higher value that gave more spatially defined receptive fields while remaining close to the normative-driven optimum (i.e., the prediction error remained small). For the autoencoder model, where there is a less well-defined objective value for the hyperparameters (the reconstruction error almost always improves for a less ‘constrained’ network), we chose a value for λ that gave the most spatially well-defined receptive fields and which best represented what is found in the visual cortex.”

We have also add the following sentence concerning λ and β to the Results (lines 171-172):

“Unless otherwise noted, λ was set to 10^-6^ and β to 0.1 (see Methods).”
