# Supplementary material for: Hierarchical recurrent temporal prediction as a model of the mammalian dorsal visual pathway
Source: PLoS Comput Biol. 2026 May 28;22(5):e1013138. doi: 10.1371/journal.pcbi.1013138 (PMC13252843; doi:10.1371/journal.pcbi.1013138)
Supplement: Peer review history

# Author comment: Author response to Decision Letter 0

**DOI:** 10.1371/journal.pcbi.1013138.r001

**Transfer Alert**

This paper was transferred from another journal. As a result, its full editorial history (including decision letters, peer reviews and author responses) may not be present.

18 May 2025

# Peer review: Decision Letter 0

**DOI:** 10.1371/journal.pcbi.1013138.r002

**Authors:** Daniele Marinazzo, Tim Christian Kietzmann

5 Aug 2025

PCOMPBIOL-D-25-00956

Hierarchical recurrent temporal prediction as a model of the mammalian dorsal visual pathway

PLOS Computational Biology

Dear Dr. King,

Thank you for submitting your manuscript to PLOS Computational Biology. After careful consideration, we feel that it has merit but does not fully meet PLOS Computational Biology's publication criteria as it currently stands. Therefore, we invite you to submit a revised version of the manuscript that addresses the points raised during the review process.

Please submit your revised manuscript within 60 days Oct 05 2025 11:59PM. If you will need more time than this to complete your revisions, please reply to this message or contact the journal office at ploscompbiol@plos.org. When you're ready to submit your revision, log on to https://www.editorialmanager.com/pcompbiol/ and select the 'Submissions Needing Revision' folder to locate your manuscript file.

Please include the following items when submitting your revised manuscript:

* A rebuttal letter that responds to each point raised by the editor and reviewer(s). You should upload this letter as a separate file labeled 'Response to Reviewers'. This file does not need to include responses to formatting updates and technical items listed in the 'Journal Requirements' section below.

* A marked-up copy of your manuscript that highlights changes made to the original version. You should upload this as a separate file labeled 'Revised Manuscript with Track Changes'.

* An unmarked version of your revised paper without tracked changes. You should upload this as a separate file labeled 'Manuscript'.

If you would like to make changes to your financial disclosure, competing interests statement, or data availability statement, please make these updates within the submission form at the time of resubmission. Guidelines for resubmitting your figure files are available below the reviewer comments at the end of this letter

We look forward to receiving your revised manuscript.

Kind regards,

Tim Christian Kietzmann, Dr. rer. nat.

Academic Editor

PLOS Computational Biology

Daniele Marinazzo

Section Editor

PLOS Computational Biology

**Journal Requirements:**

1) Please upload all main figures as separate Figure files in .tif or .eps format. For more information about how to convert and format your figure files please see our guidelines:

https://journals.plos.org/ploscompbiol/s/figures

2) Please ensure that the funders and grant numbers match between the Financial Disclosure field and the Funding Information tab in your submission form. Note that the funders must be provided in the same order in both places as well. State the initials, alongside each funding source, of each author to receive each grant. For example: "This work was supported by the National Institutes of Health (####### to AM; ###### to CJ) and the National Science Foundation (###### to AM).".

If you did not receive any funding for this study, please simply state: u201cThe authors received no specific funding for this work.u201d

**Reviewers' comments:**

Reviewer's Responses to Questions

**Comments to the Authors:**

**Please note here if the review is uploaded as an attachment.**

Reviewer #1: In this manuscript, Klavinskis-Whiting and co-authors compare response properties of units in a model network with known properties of V1, V2, and MT neurons in macaque (and mouse). The model includes both local recurrence and inter-areal feedback and is trained to perform a hierarchical temporal prediction task on natural videos. The model recapitulates several known response properties of dorsal stream neurons, thereby offering a normative hypothesis for these properties. I enjoyed reading the paper. I think it’s great work, and the findings are important. I have a couple of suggestions for the authors to consider.

Major suggestions

- The model representation appears complete (the number of units matches the number of pixels in a single frame). Is this important for the emerging stimulus response relations? I am asking because this seems to be the case in work that is somewhat related to this work (several papers by Bruno Olshausen on learning intermediate-level representations of form and motion from natural movies). If the model used fewer or more units than this magic number, would the overall pattern of results be similar? If possible, it would be great to explore and document this.

- One interesting property of MT neurons that is not explored by the authors is the organization of their RF in the 3-D Fourier-domain. Nishimoto and Gallant (2010) have done some beautiful empirical work on this, building on Heeger and Simoncelli’s (1998) theoretical model. While there has been less exploration of V1 neurons (a little bit in Zaharia et al., 2019 - eNeuro), this is presumably a property that emerges downstream of V1. Could you include an analysis that measures the degree to which RFs are organized along a velocity-plane?

Minor suggestions

- Figure 1, final panel: if I understand correctly, feedback connections were shuffled, but M was not retrained. Isn’t it trivial that performance decreases? Shouldn’t you show that even after retraining M, performance falls short of unshuffled R and M performance?

- Figure 2A: Maybe this can be quantified? How about fitting a Gabor RF and expressing the RMSE? There will likely be a systematic shift across the hierarchy. Likewise, you could use this analysis to quantify RF size and its evolution across the hierarchy.

- Ref 22 and 34 appear to be identical. One is probably intended to be Nicole Rust’s 2006 MT paper.

- The focus of this work is entirely on single unit response properties. Perception and prediction are of course supported by neural populations. Recent work suggests that the temporal geometry of population trajectories may play a key role here. Specifically, Hénaff et al (2019, 2021) proposed that natural movies elicit neural population trajectories that are straightened compared to retinal inputs. Niu, Savin and Simoncelli (2024, NeurIPS) explored this idea in a modeling study that is somewhat related to the current study. If the authors find this interesting, they could include a paragraph in the discussion in which they make the bridge between their current study and population level representations and perhaps suggest interesting avenues for future work to explore this space.

Reviewer #2: Klavinskis-Whiting et al. propose an artificial neural network (ANN) model of the dorsal visual stream in the brain. The model relies primarily on unsupervised hierarchical predictive training and incorporates architectural components such as feedback connections. The authors provide evidence that these elements are necessary for capturing certain functional properties of the dorsal pathway. In particular, they demonstrate their model's capability in reproducing frequency tuning, surround suppression, and pattern motion selectivity across dorsal areas of the monkey visual system. Additionally, through representational similarity analysis, they benchmark their model against several baseline models in terms of similarity with the mouse visual cortex. The paper is very well written and presents a compelling computational model of the dorsal visual stream. I am generally very supportive of the publication of this paper, but I do have some comments and questions that I detail below.

Major Comments:

- Could the results section include more detailed explanations of the model architecture and modules? The G modules specifically remain somewhat obscure without consulting the methods section, and providing key architectural insights in the results would improve accessibility.

- Prior works on using ANNs in modeling the dorsal pathway is lacking, especially those that have demonstrated similar neural phenomena (particularly pattern motion tuning) using different architectures and/or loss functions (DorsalNet: Mineault et al., NeurIPS, 2021, MotionNet: Rideaux and Welchman, Journal of Neuroscience, 2020). A comparison with existing models could strengthen the contribution.

- The explanations surrounding Bakhtiari et al. citation contains some inaccuracies. I am very familiar with that work, so I know that the ResNet backbones in that study were actually trained from scratch using contrastive self-supervised learning -- not pre-trained architectures. Also, in general, I don’t agree with the authors that previous contrastive models mainly relied on pretrained ResNets.

- Are the baseline models equalized in terms of parameter count? Removing components like feedback connections would decrease parameter count, which may affect the interpretation of results. The authors might consider providing comparisons with parameter-equalized models, or alternatively, could clearly report the parameter counts for each model and discuss why differences in parameter count are not expected to influence the results.

- For the memory experiments, would it be possible to train a decoder on frame 0 and test on n-back frames? This approach would provide a more precise measure of information retention over time.

- The representational similarity analysis with the mouse visual system is very informative. Could the authors better justify the choice of mouse visual areas to represent the dorsal pathway? There is some disagreement in the literature regarding which areas should be included in specific visual pathways in mice (e.g., Yu et al., Current Biology, 2022, Sit and Goard, Nature Communication, 2020) as the distinction isn't as clearly established as in primates. Additionally, the hierarchical organization among these areas remains unclear, making direct comparisons with macaque V1 and MT potentially problematic. It might be worth considering a broader examination of mouse visual areas (beyond LM and RL) independently. Notably, pattern neurons have been reported even in area LM (Matteucci et al., Science Advances, 2023), which may blur the functional boundaries between areas in the mouse visual system.

- How much might the specific training video content have influenced the results? Testing with alternative video datasets could help establish whether the findings are driven by the objective function and architecture rather than training data characteristics. This consideration is particularly important given that with unsupervised predictive training, the visual features that form the model's representations are expected to be influenced by the statistical properties of the training data. Could the authors comment on the potential impact of training data selection on their findings? It would also be helpful to include representative frames from the training videos in the supplementary materials.

- Regarding the feedback effects on plaid responses, is it clear that neurons actually convert to component-selective responses? The removal of feedback seems to disrupt tuning properties more generally, and the pattern index may not adequately capture these effects (e.g., compare panels 3Bi vs 3Fii).

Minor comments:

- Why not include spatial and temporal frequency tuning comparisons in the model evaluations? This seems particularly relevant given the proposed model's difficulty with spatial frequency patterns.

- Could the authors clarify how cycles per degree are transformed to cycles per pixel in the tuning analyses? This would help with proper comparison to existing data.

- Can the authors explain what happened when feedback was removed in the autoencoder model? Why did all responses drop to near zero?

Reviewer #3: ======. Summary ======

The authors present a biologically inspired deep network in which each layer is trained to predict the future activity of the layer below. They compare this hierarchical predictive model to two alternatives: (i) a model without feedback connections, and (ii) a model trained to predict the current rather than future input (i.e., a deep autoencoder). They find that the predictive model captures various features of visual cortical responses—such as motion tuning, surround suppression, and responses to overlapping plaids—more accurately than the alternative models.

=======Strengths ======

* The research question is timely and of clear relevance to the field.

* The proposed approach is well-motivated and the methodology appears generally sound.

* The conclusion—that the predictive model better accounts for a range of visual neuronal properties than the controls—is supported by the simulation results.

Overall, I consider this to be a strong paper, and I recommend publication pending minor revisions. I do, however, have several questions that I believe the authors should address:

=======Major Questions=======

1. **Interpretability of the Results**

The results are primarily correlational: the predictive model shows better alignment with biological data, but the underlying reasons remain unclear. Can the authors provide some theoretical insight into why the predictive model better accounts for effects like surround suppression, as compared to the autoencoder trained on present input? This is important for interpreting whether the observed behaviors are intrinsic to hierarchical predictive coding architectures, or whether they might result from implementation-specific choices (e.g., the choice of loss function, nonlinearity, enforcement of Dale’s law, etc.). While this is a challenging question, some analysis or discussion along these lines would significantly strengthen the paper.

2. **Interpolation Between Architectures**

Can the authors explore a continuous interpolation between the predictive and autoencoding models—for example, by gradually shifting the prediction target from the present to various future time points (in the multi-frame setting)? Such an approach might help clarify the functional differences between the two models.

3. **Stimulus Choice in the Multi-frame Setting**

In the multi-frame experiments, the authors restrict themselves to a simplified moving bar stimulus. Why not evaluate performance on naturalistic movie stimuli as well? This would seem a natural extension, and might yield more informative or generalizable insights.

Minor comments/Questions

- How does the comparison between predictive network and biological detail depend on parameters of objective function such as beta (which determines relative weighting to each layer in the network) and lambda (the L1 norm)? If effort was made to tune these, so as to best fit biological data, was comparable effort made for the controls?

- I was confused by the role of beta. The authors claim in line 143 that "A higher beta value indicates a greater

146 weighting applied to higher groups,". Doesn't this imply that small beta corresponds to the network being more 'feed-forward' since lower layers will receive relatively less input from higher groups? This would seem to contrast their statements in lines 148-151.

- In the initial presentation of the model (lines 117-141) I felt that there should have been more description of model details. For example, without going to the methods section we don't know if it is a rate or spiking model, what is the non-linearity, how the learning works etc. I appreciate that these details are in the methods, but since this is at the end, I think the results should have more details, otherwise the reader has to constantly refer back and forward to the methods, which is difficult.

- I think there could be more description about what are the bottlenecks/constraints in the model, for example the L1 norm. Without these, there is nothing to stop e.g. the 2nd layer just reproducing the activity of the 1st layer identically (which allows best prediction since no information is lost). Thus, given the key role of constraints/costs I think they're relatively glossed over in the main paper.

- The authors emphasize that the network satisfies certain biological constraints, such as Dale’s law. What is the impact of this constraint on the results? Would the model's behavior or predictive performance change significantly if this constraint were relaxed?

======= Minor Comments and Questions=====

1. **Sensitivity to Hyperparameters**

How does the comparison between the predictive network and biological data depend on the parameters of the objective function, such as *β* (which controls the relative weighting of each layer) and *λ* (the L1 norm coefficient)? If the authors made efforts to tune these parameters for the predictive model to best fit biological data, was a comparable effort made for the control models?

2. **Clarification on Role of β**

The role of *β* is somewhat unclear. On line 143, the authors state that “a higher beta value indicates a greater weighting applied to higher groups.” This would seem to imply that smaller *β* values make the network more feedforward, as lower layers receive proportionally less influence from higher ones. However, this appears to contrast with the interpretation in lines 148–151. Could the authors clarify this apparent contradiction?

3. **Lack of Model Detail in Initial Presentation**

In the initial description of the model (lines 117–141), important architectural and implementation details are missing. For example, it is not clear whether the model is a rate-based or spiking network, what the nonlinearity is, or how learning is implemented. While these details are included later in the Methods section, the lack of information in the main text makes the Results difficult to follow without frequent back-and-forth reference to the Methods. Including a brief summary of key model features earlier in the text would improve clarity.

4. **Underemphasis of Architectural Constraints**

The paper would benefit from a more explicit discussion of the architectural constraints that limit the model’s representational capacity—such as the L1 sparsity penalty. Without such constraints, for example, the second layer could in principle replicate the activity of the first layer exactly, achieving perfect prediction with no information loss. Since the model’s behavior depends critically on these constraints, it would be helpful if the main text gave them greater emphasis and clarified their functional role.

5. **Impact of Dale’s Law**

The authors highlight that the model enforces Dale’s law. However, it remains unclear what effect this constraint has on the results. Would the behavior or predictive performance of the model change meaningfully if Dale’s law were removed? Some comment or ablation analysis would be valuable here.

**********

**Have the authors made all data and (if applicable) computational code underlying the findings in their manuscript fully available?**

The PLOS Data policy requires authors to make all data and code underlying the findings described in their manuscript fully available without restriction, with rare exception (please refer to the Data Availability Statement in the manuscript PDF file). The data and code should be provided as part of the manuscript or its supporting information, or deposited to a public repository. For example, in addition to summary statistics, the data points behind means, medians and variance measures should be available. If there are restrictions on publicly sharing data or code —e.g. participant privacy or use of data from a third party—those must be specified.

Reviewer #1: Yes

Reviewer #2: **No:**As mentioned in the manuscript: "All modeling and analysis code written in support of this publication will be made

publicly available on a GitHub repository at the time of publication (DOI to be

assigned)."

Reviewer #3: **No:**They state that the code supporting the paper will be made available on acceptance.

**********

PLOS authors have the option to publish the peer review history of their article (what does this mean?). If published, this will include your full peer review and any attached files.

If you choose “no”, your identity will remain anonymous but your review may still be made public.

**Do you want your identity to be public for this peer review?** For information about this choice, including consent withdrawal, please see our Privacy Policy.

Reviewer #1: No

Reviewer #2: No

Reviewer #3: No

[NOTE: If reviewer comments were submitted as an attachment file, they will be attached to this email and accessible via the submission site. Please log into your account, locate the manuscript record, and check for the action link "View Attachments". If this link does not appear, there are no attachment files.]

**Figure resubmission:**

While revising your submission, please upload your figure files to the Preflight Analysis and Conversion Engine (PACE) digital diagnostic tool, https://pacev2.apexcovantage.com/. PACE helps ensure that figures meet PLOS requirements. To use PACE, you must first register as a user. Registration is free. Then, login and navigate to the UPLOAD tab, where you will find detailed instructions on how to use the tool. If you encounter any issues or have any questions when using PACE, please email PLOS at figures@plos.org. Please note that Supporting Information files do not need this step. If there are other versions of figure files still present in your submission file inventory at resubmission, please replace them with the PACE-processed versions.

**Reproducibility:**

To enhance the reproducibility of your results, we recommend that authors of applicable studies deposit laboratory protocols in protocols.io, where a protocol can be assigned its own identifier (DOI) such that it can be cited independently in the future. Additionally, PLOS ONE offers an option to publish peer-reviewed clinical study protocols. Read more information on sharing protocols at https://plos.org/protocols?utm_medium=editorial-email&utm_source=authorletters&utm_campaign=protocols

# Author comment: Author response to Decision Letter 1

**DOI:** 10.1371/journal.pcbi.1013138.r003

4 Feb 2026

Attachment Submitted filename: Response to reviewer comments final.docx

# Peer review: Decision Letter 1

**DOI:** 10.1371/journal.pcbi.1013138.r004

**Authors:** Daniele Marinazzo, Tim Christian Kietzmann

9 Mar 2026

PCOMPBIOL-D-25-00956R1

Hierarchical recurrent temporal prediction as a model of the mammalian dorsal visual pathway

PLOS Computational Biology

Dear Dr. King,

Thank you for submitting your manuscript to PLOS Computational Biology. Two reviewers were happy with the revisions you provided, and only a smaller comment remains to be addressed by a third reviewer. Therefore, we invite you to submit a revised version of the manuscript that addresses the points raised during the review process.

Please submit your revised manuscript by May 09 2026 11:59PM. If you will need more time than this to complete your revisions, please reply to this message or contact the journal office at ploscompbiol@plos.org. When you're ready to submit your revision, log on to https://www.editorialmanager.com/pcompbiol/ and select the 'Submissions Needing Revision' folder to locate your manuscript file.

Please include the following items when submitting your revised manuscript:

* A letter that responds to each point raised by the editor and reviewer(s). You should upload this letter as a separate file labeled 'Response to Reviewers'. This file does not need to include responses to formatting updates and technical items listed in the 'Journal Requirements' section below.

* A marked-up copy of your manuscript that highlights changes made to the original version. You should upload this as a separate file labeled 'Revised Manuscript with Track Changes'.

* An unmarked version of your revised paper without tracked changes. You should upload this as a separate file labeled 'Manuscript'.

If you would like to make changes to your financial disclosure, competing interests statement, or data availability statement, please make these updates within the submission form at the time of resubmission. Guidelines for resubmitting your figure files are available below the reviewer comments at the end of this letter.

We look forward to receiving your revised manuscript.

Kind regards,

Tim Christian Kietzmann, Dr. rer. nat.

Academic Editor

PLOS Computational Biology

Daniele Marinazzo

Section Editor

PLOS Computational Biology

**Journal Requirements:**

1) Regarding Figure 1B, thank you for stating that it "includes an image from the open repository Zenodo (DOI 10.5281/zenodo.3926116), which has a CC BY 4.0 licence." Please ensure that the source details are included in the figure legend.

Note: If the reviewer comments include a recommendation to cite specific previously published works, please review and evaluate these publications to determine whether they are relevant and should be cited. There is no requirement to cite these works unless the editor has indicated otherwise.

**Reviewers' comments:**

Reviewer's Responses to Questions

Reviewer #1: The authors have addressed my concerns. It is a beautiful piece of work.

Reviewer #2: I thank the authors for their thorough revisions. I have a remaining question regarding the role of feedback in pattern motion selectivity.

Specifically, there appears to be a discrepancy between the results in Figure 3F and those in the new Supplementary Figure S7. In Figure 3F, the tuning curves become notably noisy following feedback removal, suggesting a general degradation of motion selectivity rather than a clear shift toward component selectivity. In contrast, the tuning curves in Figure S7 align much more closely with the paper’s hypothesis. Could the authors clarify why these two sets of results differ? Furthermore, given that Figure S7 provides a clearer demonstration of the proposed mechanism, I would encourage the authors to explain why Figure 3F was selected for the main text instead.

Reviewer #3: I commend the authors on the hard work they have put in to addressing my, and the other reviewers' comment. I recommend for publication.

I just have a couple of tiny points regarding their answers to my comments:

(1) For the multi-frame setting the authors responded that they found no improved multi-frame prediction for non-zero beta.

(2) Regarding parameter tuning the authors have added "“For the temporal prediction model and the control model, some exploration of hyperparameters λ and β was performed to find those values that produced biological realistic receptive fields, with the temporal prediction also having the additional independent criterion that prediction error remained small.” Could they be more explicit about what objective criteria were used to choose their parameters in each case (and including the controls)?

**********

**Have the authors made all data and (if applicable) computational code underlying the findings in their manuscript fully available?**

The PLOS Data policy requires authors to make all data and code underlying the findings described in their manuscript fully available without restriction, with rare exception (please refer to the Data Availability Statement in the manuscript PDF file). The data and code should be provided as part of the manuscript or its supporting information, or deposited to a public repository. For example, in addition to summary statistics, the data points behind means, medians and variance measures should be available. If there are restrictions on publicly sharing data or code —e.g. participant privacy or use of data from a third party—those must be specified.

Reviewer #1: Yes

Reviewer #2: Yes

Reviewer #3: Yes

**********

PLOS authors have the option to publish the peer review history of their article (what does this mean?). If published, this will include your full peer review and any attached files.

If you choose “no”, your identity will remain anonymous but your review may still be made public.

**Do you want your identity to be public for this peer review?** For information about this choice, including consent withdrawal, please see our Privacy Policy.

Reviewer #1: No

Reviewer #2: No

Reviewer #3: No

[NOTE: If reviewer comments were submitted as an attachment file, they will be attached to this email and accessible via the submission site. Please log into your account, locate the manuscript record, and check for the action link "View Attachments". If this link does not appear, there are no attachment files.]

**Figure resubmission:**

While revising your submission, we strongly recommend that you use PLOS’s NAAS tool (https://ngplosjournals.pagemajik.ai/artanalysis) to test your figure files. NAAS can convert your figure files to the TIFF file type and meet basic requirements (such as print size, resolution), or provide you with a report on issues that do not meet our requirements and that NAAS cannot fix.

After uploading your figures to PLOS’s NAAS tool - https://ngplosjournals.pagemajik.ai/artanalysis, NAAS will process the files provided and display the results in the "Uploaded Files" section of the page as the processing is complete. If the uploaded figures meet our requirements (or NAAS is able to fix the files to meet our requirements), the figure will be marked as "fixed" above. If NAAS is unable to fix the files, a red "failed" label will appear above. When NAAS has confirmed that the figure files meet our requirements, please download the file via the download option, and include these NAAS processed figure files when submitting your revised manuscript.

**Reproducibility:**

To enhance the reproducibility of your results, we recommend that authors of applicable studies deposit laboratory protocols in protocols.io, where a protocol can be assigned its own identifier (DOI) such that it can be cited independently in the future. Additionally, PLOS ONE offers an option to publish peer-reviewed clinical study protocols. Read more information on sharing protocols at https://plos.org/protocols?utm_medium=editorial-email&utm_source=authorletters&utm_campaign=protocols

# Author comment: Author response to Decision Letter 2

**DOI:** 10.1371/journal.pcbi.1013138.r005

6 May 2026

Attachment Submitted filename: response to reviewers final.docx

# Editor report: Decision Letter 2

**DOI:** 10.1371/journal.pcbi.1013138.r006

**Authors:** Daniele Marinazzo, Tim Christian Kietzmann

7 May 2026

Dear Professor King,

We are pleased to inform you that your manuscript 'Hierarchical recurrent temporal prediction as a model of the mammalian dorsal visual pathway' has been provisionally accepted for publication in PLOS Computational Biology.

Before your manuscript can be formally accepted you will need to complete some formatting changes, which you will receive in a follow up email. A member of our team will be in touch with a set of requests.

Please note that your manuscript will not be scheduled for publication until you have made the required changes, so a swift response is appreciated.

IMPORTANT: The editorial review process is now complete. PLOS will only permit corrections to spelling, formatting or significant scientific errors from this point onwards. Requests for major changes, or any which affect the scientific understanding of your work, will cause delays to the publication date of your manuscript.

Should you, your institution's press office or the journal office choose to press release your paper, you will automatically be opted out of early publication. We ask that you notify us now if you or your institution is planning to press release the article. All press must be co-ordinated with PLOS.

Thank you again for supporting Open Access publishing; we are looking forward to publishing your work in PLOS Computational Biology.

Best regards,

Tim Christian Kietzmann, Dr. rer. nat.

Academic Editor

PLOS Computational Biology

Daniele Marinazzo

Section Editor

PLOS Computational Biology

***********************************************************

# Editor report: Acceptance letter

**DOI:** 10.1371/journal.pcbi.1013138.r007

**Authors:** Daniele Marinazzo, Tim Christian Kietzmann

PCOMPBIOL-D-25-00956R2

Hierarchical recurrent temporal prediction as a model of the mammalian dorsal visual pathway

Dear Dr King,

I am pleased to inform you that your manuscript has been formally accepted for publication in PLOS Computational Biology. Your manuscript is now with our production department and you will be notified of the publication date in due course.

The corresponding author will soon be receiving a typeset proof for review, to ensure errors have not been introduced during production. Please review the PDF proof of your manuscript carefully, as this is the last chance to correct any errors. Please note that major changes, or those which affect the scientific understanding of the work, will likely cause delays to the publication date of your manuscript.

Soon after your final files are uploaded, unless you have opted out, the early version of your manuscript will be published online. The date of the early version will be your article's publication date. The final article will be published to the same URL, and all versions of the paper will be accessible to readers.

For Research, Software, and Methods articles, you will receive an invoice from PLOS for your publication fee after your manuscript has reached the completed accept phase. If you receive an email requesting payment before acceptance or for any other service, this may be a phishing scheme. Learn how to identify phishing emails and protect your accounts at https://explore.plos.org/phishing.

Thank you again for supporting PLOS Computational Biology and open-access publishing. We are looking forward to publishing your work!

With kind regards,

Aiswarya Satheesan

PLOS Computational Biology | Carlyle House, Carlyle Road, Cambridge CB4 3DN | United Kingdom ploscompbiol@plos.org | Phone +44 (0) 1223-442824 | ploscompbiol.org | @PLOSCompBiol
